# NOX2 deficiency exacerbates lupus nephritis through activation of NLRP3 inflammasome in neutrophils

**DOI:** 10.1186/s12929-026-01287-2

**Published:** 2026-08-19

**Authors:** Chun-Hsin Wu, Fang-Yu Lin, Miao-Shan Lin, Tzu-Yi Chan, Ying-Ren Chen, Hui-Ching Cheng, Chao-Kai Hsu, Chih-An Chen, Peng-Chieh Chen, Siao-Muk Cheng, Daw-Yang Hwang, Junne-Ming Sung, Yau-Sheng Tsai, Pin Ling, Chi-Chang Shieh

**Affiliations:** 1 Institute of Clinical Medicine, College of Medicine, National Cheng Kung University, No. 35, Xiaodong Road, Tainan City, 704 Taiwan; 2 Division of Allergy, Immunology and Rheumatology, Department of Internal Medicine, National Cheng Kung University Hospital, College of Medicine, National Cheng Kung University, Tainan, Taiwan; 3 Department of Pathology, National Cheng Kung University Hospital, College of Medicine, National Cheng Kung University, Tainan, Taiwan; 4 Department of Dermatology, National Cheng Kung University Hospital, College of Medicine, National Cheng Kung University, Tainan, Taiwan; 5 Department of Pediatrics, National Cheng Kung University Hospital, College of Medicine, National Cheng Kung University, Tainan, Taiwan; 6 National Institute of Cancer Research, National Health Research Institutes, Tainan, Taiwan; 7 Division of Nephrology, Department of Internal Medicine, National Cheng Kung University Hospital, College of Medicine, National Cheng Kung University, Tainan, Taiwan; 8 Department of Microbiology and Immunology, College of Medicine, National Cheng Kung University, Tainan, Taiwan

**Keywords:** Lupus nephritis, Neutrophils, NOX2, Reactive oxygen species, NLRP3, IL-1β

## Abstract

**Background:**

Lupus nephritis (LN) is a severe manifestation of systemic lupus erythematosus (SLE) associated with significant morbidity. Although reduced production of reactive oxygen species (ROS) by neutrophils correlates with severe SLE, the specific mechanisms linking ROS deficiency to heightened renal inflammation remain unknown. We aimed to elucidate the role of NOX2-derived ROS in LN pathogenesis and identify potential therapeutic targets.

**Methods:**

We conducted an in vivo study using a pristane-induced lupus model in *Ncf1*^−/−^ (NOX2-deficient) mice and wild-type controls. We assessed LN severity and characterized renal immune infiltration using flow cytometry and single-cell RNA sequencing (scRNA-seq). We performed transcriptomic analysis to evaluate the function of NOX2-deficient neutrophils. Finally, we tested the therapeutic efficacy of an IL-1 receptor antagonist in ameliorating disease severity in the NOX2-deficient mice.

**Results:**

NOX2 deficiency exacerbated LN severity compared to wild-type controls, demonstrated by increased serum anti-dsDNA antibody titers and worsened LN scores. Through scRNA-seq, we identified a distinct, activated neutrophil subset in *Ncf1*^−/−^ mice featuring a robust interferon signature and high *Nlrp3* expression. Transcriptomic analysis confirmed the upregulation of core NLRP3 pathway within these cells. Crucially, treating NOX2-deficient mice with an IL-1 receptor antagonist reduced LN activity scores.

**Conclusion:**

Our results showed that NOX2 deficiency was associated with the expansion of a highly inflammatory renal neutrophil subset that may contribute to aggravated renal inflammation. These findings suggest that NOX2 functions as a negative regulator of the NLRP3 inflammasome and IL-1β blockade represents a promising precision therapeutic strategy for patients with LN who exhibit impaired ROS production.

**Supplementary Information:**

The online version contains supplementary material available at 10.1186/s12929-026-01287-2.

## Introduction

Systemic lupus erythematosus (SLE) is a heterogeneous autoimmune disorder characterized by the loss of tolerance to nuclear self-antigens, leading to the production of autoantibodies and the subsequent deposition of immune complexes in various organs [1]. Among the clinical manifestations of SLE, lupus nephritis (LN) represents a major determinant of morbidity and mortality, affecting approximately 50% to 60% of patients [2]. The pathogenesis of LN involves a complex interplay between innate and the adaptive immune system. In particular, the infiltration of innate immune cells, specifically neutrophils, macrophages, and dendritic cells into renal tissue mediates local inflammation and tissue injury. While glomerular neutrophil accumulation and NET deposition are features of active, proliferative LN, the precise mechanisms linking specific neutrophil subpopulations to early renal injury remain incompletely understood [3].

A common component of the innate immune response is the production of reactive oxygen species (ROS) by the nicotinamide adenine dinucleotide phosphate (NADPH) oxidase-2 (NOX2) complex. Historically, ROS were viewed primarily as pro-inflammatory mediators of oxidative tissue damage. However, the clinical phenotype of chronic granulomatous disease (CGD), an immunodeficiency caused by loss-of-function mutations in NOX2 subunits, has revealed a paradoxical role for ROS in immune regulation [4]. Patients with CGD exhibit a marked predisposition to autoimmune disorders, most notably SLE and inflammatory arthritis [5]. This protective role of functional NOX2 is further supported by genetic association studies identifying the *NCF1* gene, which encodes the p47phox regulatory subunit of NOX2, as a significant susceptibility locus for SLE [6]. Specifically, a missense single nucleotide polymorphism known as *NCF1*−339 is strongly associated with reduced ROS production and an increased risk of developing SLE, particularly in Asian populations [7]. These findings suggest that a deficiency in NOX2-derived ROS disrupts a critical immune checkpoint, thereby predisposing the host to autoimmunity. Furthermore, our previous work demonstrated that a loss-of-function mutation in *Ncf1*, exacerbates immune-mediated arthritis via mechanisms involving neutrophil dysregulation and elevated IL-1β production [8, 9].

A major pathway of IL-1β production involves the NOD-like receptor family pyrin domain-containing 3 (NLRP3) inflammasome activation in innate immune cells, particularly neutrophils [10]. Previous studies have demonstrated that the expression of IL-1β and NLRP3 is significantly elevated in the renal tissue of patients with active LN [11]. However, the precise relationship between NOX2 deficiency and NLRP3 inflammasome hyperactivation in the specific context of renal inflammation remains a subject of ongoing investigation. While recent evidence in human monocytes suggests that NOX2 deficiency enhances the transcriptional priming of NLRP3 and IL-1β via an IL-1β-dependent activation of the NF-κB signaling pathway [12], it is unclear whether this mechanism drives the pathogenesis of LN, particularly by modulating the activity of renal neutrophils.

Given that our prior studies revealed that NOX2 deficiency exacerbates immune-mediated arthritis through dysregulated neutrophils characterized by hyperinflammatory IL-1β responses [8, 9], we hypothesize that a lack of NOX2-derived ROS in renal neutrophils promotes the NLRP3 inflammasome, leading to excessive IL-1β production and aggravates glomerular inflammation. In the present study, we utilized a pristane-induced murine model of lupus (PIL) to elucidate the pathogenic link between NOX2 deficiency and LN severity. We aimed to characterize the transcriptomic profile of NOX2-deficient renal neutrophils and evaluated whether targeting the NLRP3-IL-1β axis offers a therapeutic strategy to mitigate renal injury in the setting of reduced oxidative stress.

## Material and methods

### Animal model

Eight-week-old female BALB/c mice were used. BALB/c. *Ncf1*^<m1J>/J^ mice were established by backcrossing onto the BALB/c background for ten generations, and *Ncf1* deficiency was confirmed by genotyping. Murine lupus was induced by intraperitoneal administration of pristane (500 µL; Sigma-Aldrich) at 8 weeks of age and again 2 weeks later. Mice were euthanized 12 weeks after pristane administration. Animals were housed under specific pathogen-free conditions with a 12 h light/12 h dark cycle and controlled temperature and humidity at the Institutional Animal Care and Use Committee-approved facility of National Cheng Kung University.

### Renal pathology: histopathological assessment and activity index score

For the assessment of renal pathology, kidneys were harvested following perfusion with phosphate-buffered saline (PBS) and immediately fixed with 10% buffered formalin (Sigma). Tissue sections were subsequently cut and stained with hematoxylin and eosin (H&E). Nephritis activity was quantified using a composite scoring system based on the modified NIH lupus nephritis activity and chronicity index [13] and murine urinary pathology criteria described by Frazier et al. [14]. Four specific pathologic features were evaluated: (1) endocapillary hypercellularity, (2) neutrophils/karyorrhexis, (3) interstitial inflammation, and (4) mesangial proliferation. Endocapillary hypercellularity, neutrophils/karyorrhexis, and interstitial inflammation (defined by leukocytes within the cortex) were each scored according to the extent of involvement: < 25% (1+), 25%–50% (2+), or > 50% (3+). Mesangial proliferation, characterized by hyperplasia, hypertrophy, and increased mesangial matrix, was graded on a scale of 0 to 4. A total of 40 glomeruli and 10 cortical fields were evaluated per kidney sample, and the activity index score was recorded as the sum of the scores for these four parameters.

### Immunofluorescence staining

To assess endogenous IgG deposition within the glomeruli, immunofluorescence staining was conducted; comprehensive experimental procedures are provided in the Supplementary Materials and Methods.

### Measurement of cytokines and autoantibodies

Murine IFN-α, IL-1β, and IL-6 levels were quantified using Mouse ELISA MAX Deluxe Sets (BioLegend). Serum anti-dsDNA IgG levels were measured using an anti-dsDNA IgG ELISA kit (Cat# MBS269288; BioSource). All assays were performed according to the manufacturers' instructions.

### Isolation of cells from kidney

Mouse kidneys were minced into small fragments and digested in RPMI-1640 medium containing collagenase D (1.4 mg/mL; Roche) and DNase I (0.1 mg/mL; Sigma-Aldrich) at 37 ℃ for 45 min with gentle agitation. Following digestion, the tissue suspension was mechanically dissociated by repeated pipetting and filtered through a 70-µm cell strainer. Leukocytes were further enriched using a discontinuous Percoll (GE Healthcare, Cytiva) density gradient (36% and 72%). After centrifugation, cells at the interface between 36 and 72% were collected.

### Flow cytometric analysis

The enriched leukocyte fraction was incubated with anti-mouse CD16/32 (clone 2.4G2; 553142D) at 4 ℃ for 20 min. After washing, cells were stained with fluorochrome-conjugated monoclonal antibodies against surface markers (listed below) in staining buffer at 4 ℃ for 20 min protected from light. All staining procedures were conducted in accordance with the manufacturer's instructions. A comprehensive list of the specific antibodies utilized is provided in Supplementary Table S1 in the Supplementary Materials and Methods. Flow cytometric data were acquired utilizing a FACSCanto II flow cytometer (BD Biosciences) and subsequently analyzed using FlowJo software (version 10; FlowJo LLC, Ashland, OR, USA).

### Intracellular IL-1β staining in renal neutrophils

For intracellular IL-1β staining, surface marker-stained cells were fixed and permeabilized using the BD Cytofix/Cytoperm Fixation/Permeabilization Kit (Cat. No. 554714; BD Biosciences) according to the manufacturer’s instructions. Cells were washed with BD Perm/Wash Buffer and stained with a FITC-conjugated anti-mouse pro-IL-1β monoclonal antibody (Cat. No. 11–7114-80; Thermo Fisher Scientific) diluted in BD Perm/Wash Buffer at 4 ℃ for 30 min in the dark. After washing and resuspension in staining buffer, kidney neutrophils were defined as CD45⁺CD11b⁺Ly6G⁺ cells after singlet gating, and intracellular IL-1β expression was quantified with flow cytometry as geometric mean fluorescence intensity within this population.

### Bulk RNA-sequencing

Comprehensive procedures regarding sample preparation, sequencing protocols, and data processing were detailed in the Supplementary Materials and Methods.

### Single cell RNA sequencing

Kidney single-cell suspensions were prepared as described above and used for single-cell RNA sequencing (scRNA-seq). Cells were incubated with anti-mouse CD16/32 (clone 2.4G2; 553142, BD) for 20 min, followed by surface staining with CD45 PerCP/cyanine5.5 (30F11103130, BioLegend) for 20 min. Prior to sorting, cells were passed through a 30-μm mesh and CD45⁺ cells were purified on a FACSAria cell sorter (BD Biosciences). Sorted cells were collected in RPMI supplemented with 20% FBS, washed sequentially and resuspended for fixation. 1 × 10^6 cells per sample were fixed for 22 h at 4℃, quenched, and stored at − 80 ℃ according to the 10× Genomics Fixation of Cells & Nuclei for Chromium Fixed RNA Profiling protocol (CG000478) using the Chromium Next GEM Single Cell Fixed RNA Sample Preparation Kit (PN-1000414, 10× Genomics). Fixed RNA profiling was performed using the Chromium Fixed RNA Kit, Mouse Transcriptome, 4 rxn × 16 bc (PN-1000497, 10× Genomics). Following probe hybridization, samples were pooled and processed using the Pooled Wash Workflow, and libraries were generated according to the Chromium Fixed RNA Profiling Reagent Kit protocol (CG000527, 10× Genomics, Rev D). Sequencing was performed on an Illumina NovaSeq platform with paired-end 150-bp reads.

### scRNA-seq data processing and analysis

Single-cell RNA-seq data were analyzed in R using Seurat (v4.4.2) [15]. Cells with fewer than 200 detected genes, more than 5,000 detected genes, or with > 30% mitochondrial transcripts were excluded. Doublets were identified and removed using DoubletFinder (v2.0.3) [16]. Data were log-normalized using NormalizeData, and the top 2,000 highly variable genes were identified with FindVariableFeatures. The four datasets were integrated using Seurat’s anchor-based workflow (FindIntegrationAnchors and IntegrateData), followed by scaling (ScaleData) and principal component analysis (RunPCA). The first 30 principal components were used to construct a shared nearest-neighbor graph (FindNeighbors; dims = 1–30), perform clustering (FindClusters; resolution = 0.25), and generate UMAP embeddings (RunUMAP; dims = 1–30). Detailed protocols regarding leukocytes annotation and neutrophil subgroup analysis were provided in the Supplementary Materials and Methods. Cluster marker genes were identified using default FindAllMarkers function using thresholds of average log2 fold change ≥ 0.25 and adjusted P value < 0.05. Differentially expressed genes were ranked for each cluster, and the top 10 were visualized using DoHeatmap. Cellular trajectories and pseudotime analysis were inferred utilizing the Monocle2 R package (version 2.34.0) [17]. Comprehensive methodological details regarding this analysis are outlined in the Supplementary Materials and Methods.

### Quantitative real-time PCR

Renal neutrophils were purified from the Percoll-enriched leukocyte fraction via flow cytometric sorting utilizing a FACSAria cell sorter (BD Biosciences). The specific antibodies employed for this isolation procedure are listed in Supplementary Table S1. Total RNA was extracted directly from sorted neutrophils using the Direct-zol RNA MicroPrep Kit (Zymo Research, Cat. R1050) according to the manufacturer’s instructions. cDNA was synthesized using the High-Capacity cDNA Reverse Transcription Kit (Applied Biosystems, Thermo Fisher Scientific, Cat. 4368814). Quantitative real-time PCR was performed using PowerUp SYBR Green Master Mix (Applied Biosystems, Thermo Fisher Scientific, Cat. A25741) on a StepOnePlus Real-Time PCR System (Applied Biosystems, Thermo Fisher Scientific) with gene-specific primers. Primer sequences were listed in the Supplementary Materials and Methods.

### In vivo IL-1β inhibition

Anakinra (Kineret®; 100 mg/0.67 mL) was diluted in sterile 1× PBS and administered to mice at 75 mg/kg via intraperitoneal injection. For vehicle controls, an equivalent volume of sterile 1× PBS was administered. All treatments were prepared fresh immediately prior to dosing by diluting anakinra or sterile 1× PBS to the desired working concentration.

### Statistical analysis

Data were expressed as mean ± standard error of the mean (SEM) or median with interquartile range (IQR), as deemed appropriate based on the distribution of the data. For comparisons between two groups, the unpaired Student's *t*-test was utilized for normally distributed data, while the Wilcoxon rank-sum test was employed for non-normally distributed data. For comparisons involving three or more groups, one-way analysis of variance (ANOVA) or the Kruskal–Wallis test was conducted, followed by appropriate post-hoc tests to identify specific group differences. All statistical analyses were performed using GraphPad Prism software, version 7.0 (GraphPad Software, Inc., La Jolla, CA, USA). Statistical significance was defined as a *p*-value of less than 0.05. Significance levels were denoted as follows: *p < 0.05, **p < 0.01, ***p < 0.001, and ****p < 0.0001.

## Results

### Pristane induced more severe lupus activity in NOX2-deficient mice

**Fig. 1:**
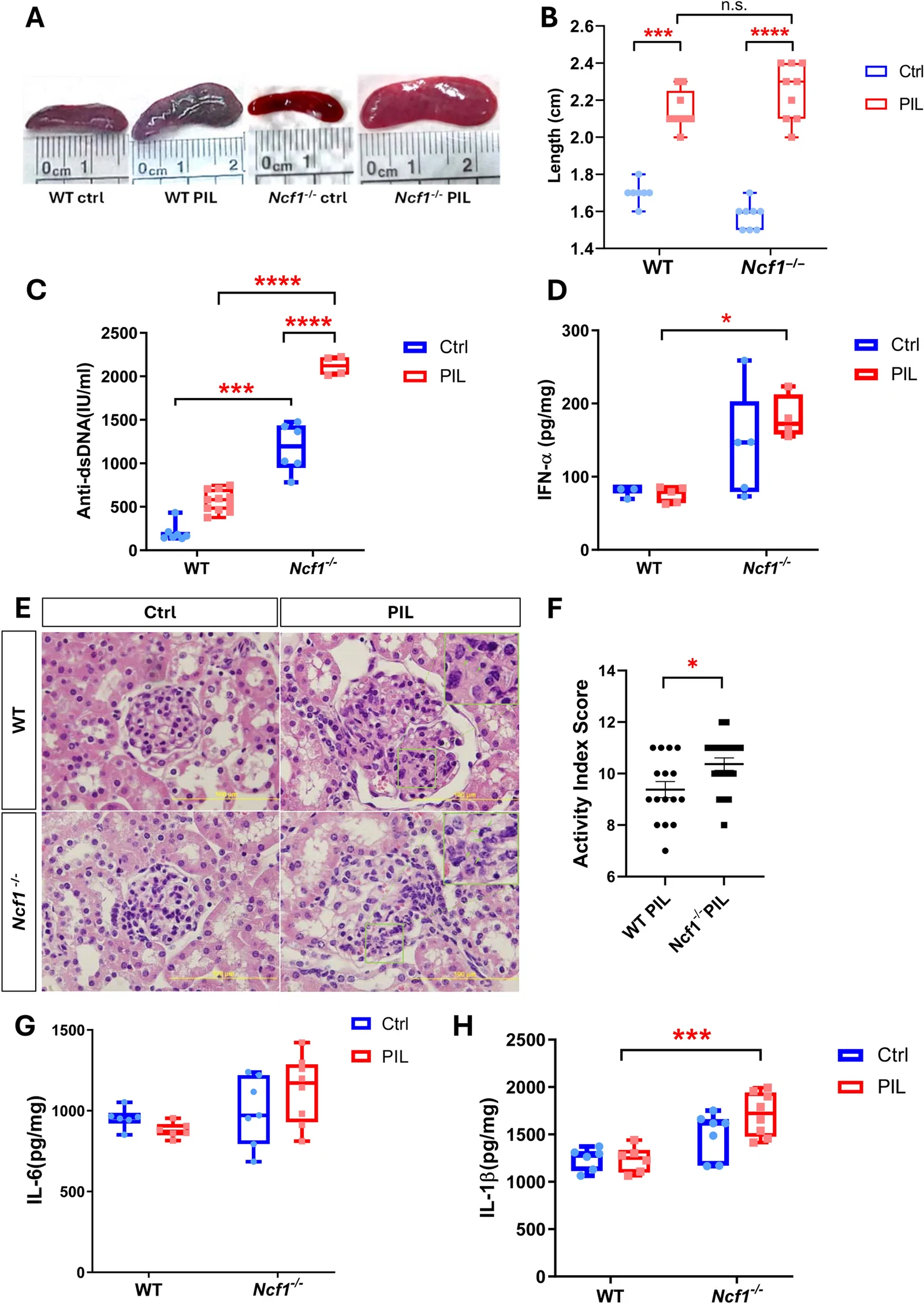
Exacerbation of autoimmunity and systemic inflammation in NOX2-deficient (*Ncf1*^*–/–*^) mice with PIL.** A** Representative images of spleens from WT control, WT PIL, *Ncf1*^–/–^ control, and *Ncf1*^–/–^ PIL mice at 12 weeks post-induction. **B** Quantification of spleen length (cm) across four experimental groups: WT control (n = 7), WT PIL (n = 9), *Ncf1*^–/–^ control (n = 8), and *Ncf1*^–/–^ PIL (n = 9). **C** Serum anti-dsDNA autoantibody levels (IU/mL) measured by ELISA: WT control (n=7), WT PIL (n = 11), *Ncf1*^–/–^ control (n = 6), and *Ncf1*^–/–^ PIL (n = 4). **D** IFN-α concentrations (pg/mg) in kidney tissue homogenates: WT control (n = 3), WT PIL (n = 5), *Ncf1*^–/–^ control (n = 5), and *Ncf1*^–/–^ PIL(n = 4). **E** Representative H&E staining of kidney sections (scale bar = 100 µm) with insets highlighting neutrophil infiltration and karyorrhexis (green arrows). **F** Lupus nephritis activity index comparing WT PIL (n = 16) and *Ncf1*^–/–^ PIL (n = 19) groups. **G**, **H** Renal tissue concentrations (pg/mg) of the pro-inflammatory cytokines IL-6 (**G**) and IL-1β (**H**): WT control (n = 6), WT PIL (n = 6), *Ncf1*^–/–^ control (n = 7), and *Ncf1*^–/–^ PIL(n = 8). Data represent the pooled median [IQR] from two independent experiments. Statistical significance was determined using the Wilcoxon rank-sum test. Asterisks indicate statistically significant differences between designated groups (*p < 0.05, ***p < 0.001, ****p < 0.0001; ns: non-significant)

To address the impact of leukocyte-produced ROS in lupus, we established an inducible murine lupus model by pristane injection in *Ncf1*^−/−^ and wild-type (WT) mice. Mice were sacrificed 12 weeks after two doses of intraperitoneal administration of pristane. Swelling of spleen was found in both WT and *Ncf1*^−/−^ lupus mice after pristane administration (Fig. 1A and 1B). Levels of serum anti-dsDNA autoantibody (Fig. 1C) and renal IFN-α (Fig. 1D) were significantly higher in *Ncf1*^−/−^ PIL compared to WT PIL. Prior to pristane administration, the baseline level of renal IFN-α was higher in *Ncf1*^*−/−*^ control mice compared to WT controls (Fig. 1D). Renal histopathology analyses revealed more severe glomerular inflammation in *Ncf1*^*−/−*^ PIL compared to WT PIL (Fig. 1E), accompanied by a significantly higher LN activity index score (Fig. 1F). Increased immunocomplexes deposition was observed in both *Ncf1*^−/−^ and WT PIL (Supplementary Figure S1). We observed that there was no significant difference between levels of IL-6 in kidney homogenates of *Ncf1*^−/−^ and WT PIL (Fig. 1G). In contrast, the levels of IL-1β in kidney homogenates were significantly higher in *Ncf1*^−/−^ PIL than in WT PIL (Fig. 1H). Similar to the trends observed with IFN-α, baseline renal IL-1β levels were also increased in *Ncf1*^*−/−*^ controls relative to WT controls (Fig. 1H). This result was in line with our previous findings that IL-1β was higher in inflamed joints of *Ncf1*^−/−^ arthritic mice. The above data suggested that the increase in IL-1β may play an important role in aggravating the severity of lupus phenotype in ROS-deficient mice.

### Differential distribution of CD45^+^ leukocytes in the kidney of wild type and NOX2-deficient lupus mice

**Fig. 2:**
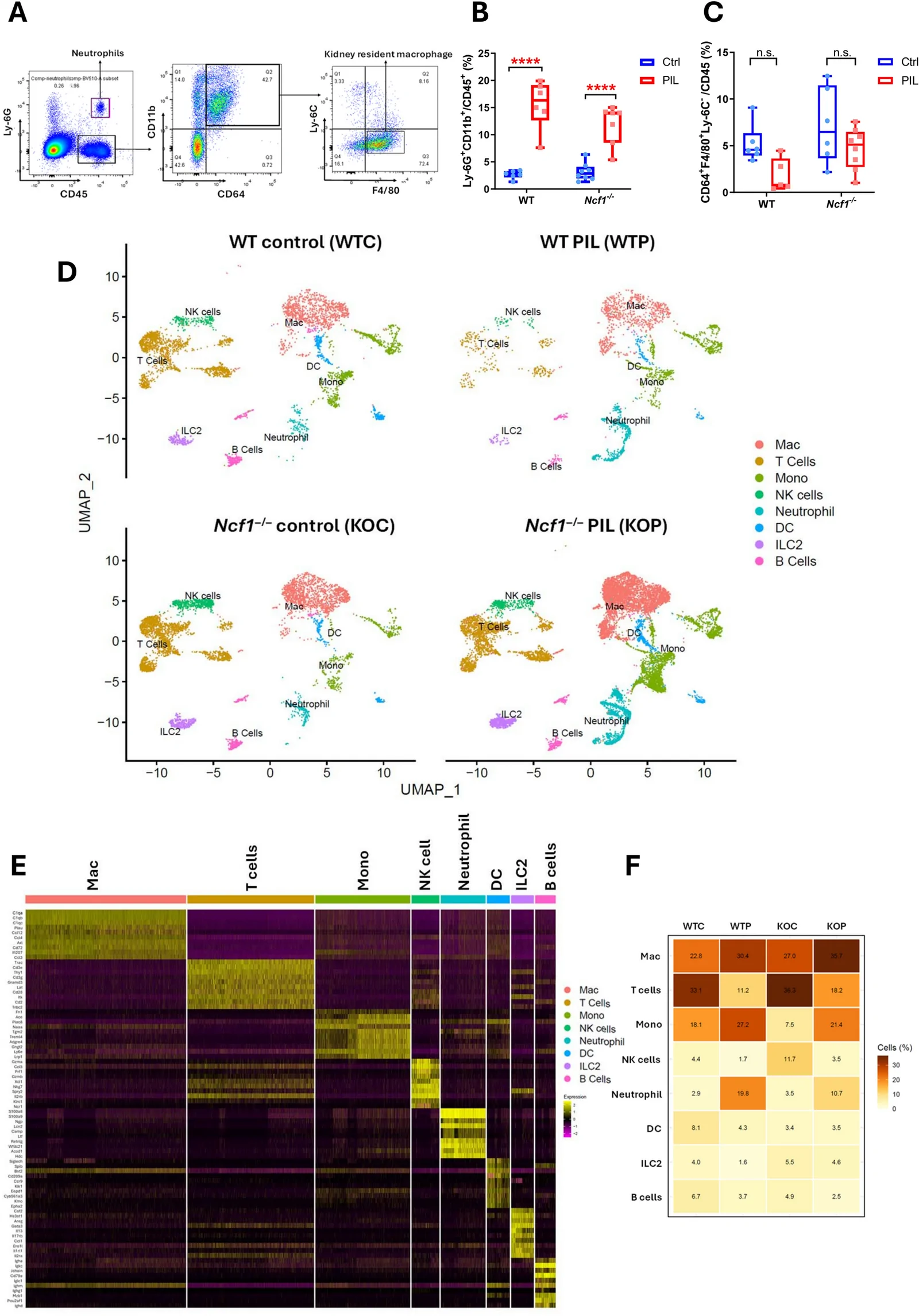
Flow cytometry and single-cell RNA sequencing show a differential distribution of renal CD45^+^ leukocytes in WT and *Ncf1*^*–/–*^ mice with PIL. **A** Representative flow cytometry gating strategy for identifying renal neutrophils (Ly6G^+^CD11b^+^) and kidney-resident macrophages (CD64^+^F4/80^+^Ly6C^−^). **B** Frequency of renal neutrophils (expressed as a percentage of total CD45^+^ leukocytes): WT control (n = 6), WT PIL (n = 6), *Ncf1*^–/–^ control (n = 8), and *Ncf1*^–/–^ PIL (n = 7) mice. **C** Frequency of kidney-resident macrophages (KRM, expressed as a percentage of total CD45^+^ leukocytes): WT control (n = 6), WT PIL (n = 6), *Ncf1*^–/–^ control (n = 6), and *Ncf1*^–/–^ PIL (n = 8). **D** UMAP visualization of renal immune cells from WT control, WT PIL, *Ncf1*^–/–^ control, and *Ncf1*^–/–^ PIL mice. **E** Heatmap showing the top marker genes for the identified cell clusters. **F** Heatmap summarizing the cell distribution (%) across the eight major immune cell clusters for each experimental group. Flow cytometry data were pooled from two independent experiments and are presented as box plots showing the median and IQR, with individual data points representing biological replicates. Statistical significance was determined by the Kruskal–Wallis test: ****p < 0.0001

In lupus nephritis, innate immune cells, including neutrophils and macrophages, respond to deposited immune complexes [18]. We first examined renal inflammation by quantifying neutrophil and macrophage frequencies via flow cytometry (Fig. 2A). This analysis revealed that pristane induction increased the frequency of renal neutrophils relative to the corresponding controls in both *Ncf1*^*−/−*^ PIL mice and WT PIL mice. The neutrophil frequencies among CD45⁺ cells were equivalent in *Ncf1*^*−/−*^ PIL and WT PIL mouse groups (Fig. 2B). In contrast, the frequency of kidney-resident macrophages (KRM, defined as CD64^+^, F4/80^+^, and Ly6C^−^ cells among CD45^+^ leukocytes) decreased in both *Ncf1*^−/−^ and WT PIL than controls (Fig. 2C). To define the impact of NOX2-derived ROS on renal immune cells, we conducted scRNA-seq on CD45⁺ leukocytes isolated from the kidneys of *Ncf1*^−/−^ and WT PIL, alongside controls. After quality control and doublet filtering, 23,010 single-cell transcriptomic results (WT control: 4,232; WT PIL: 2,401; *Ncf1*^−/−^ control: 5,187, *Ncf1*^−/−^ PIL:11,190) were obtained for further analyses using Seurat. Projection of the integrated dataset by unsupervised UMAP confirmed CD45 expression (Figure S2) and revealed 8 cell clusters (Fig. 2D and E). Next, we determined the proportion of each cell type in different experimental mice (Fig. 2F). A higher proportion of neutrophils was seen in *Ncf1*^−/−^ mice than in WT mice. Combined flow cytometric and scRNA-seq analyses revealed that NOX2-derived ROS significantly modulate intrarenal innate immune cell frequencies in murine lupus.

### Neutrophils from kidneys of NOX2 deficient mice showed an activated phenotype in the inflammasome pathway

**Fig. 3:**
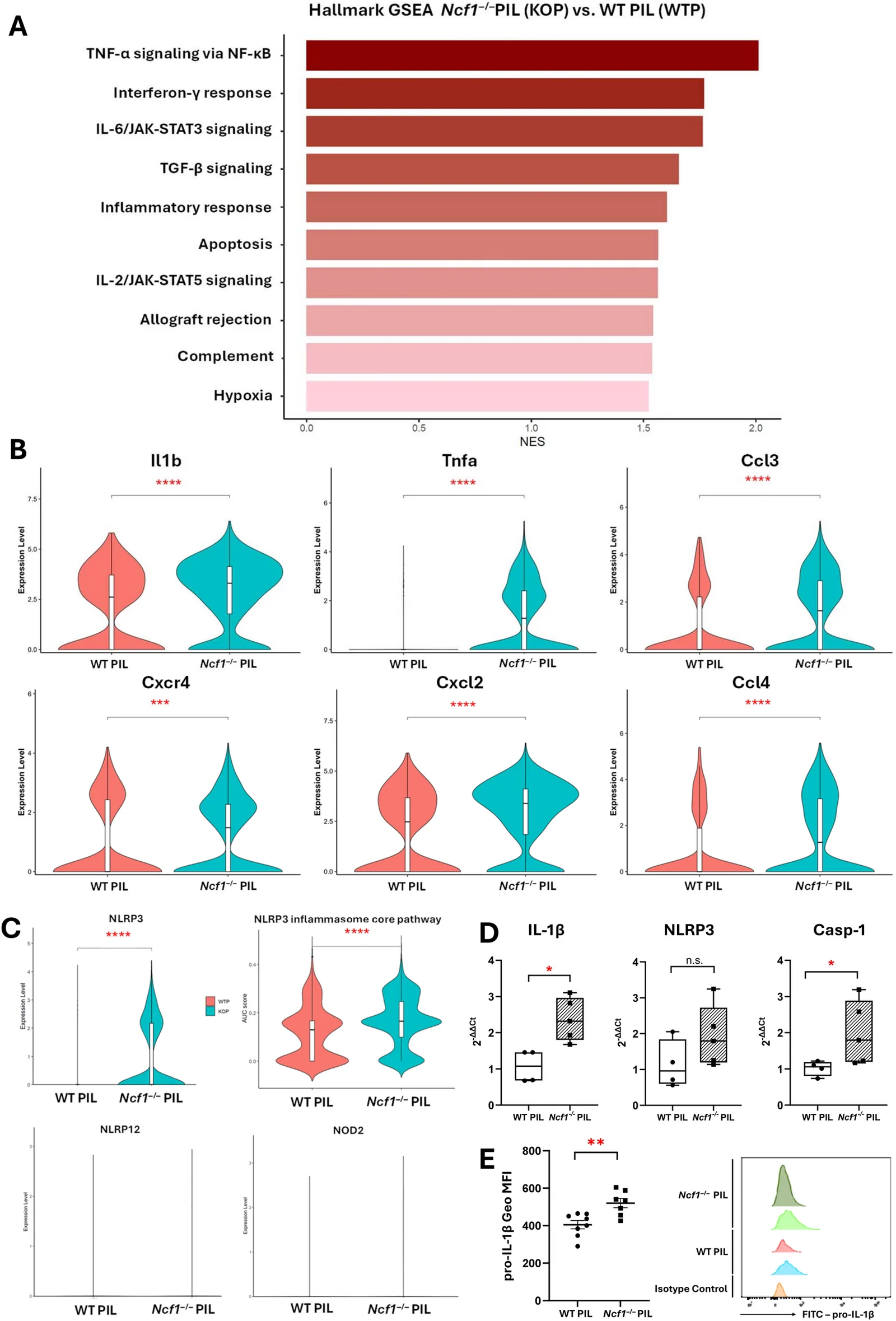
NOX2 deficiency amplifies inflammatory signaling and NLRP3 activation in renal neutrophils.** A** Bar chart showing the top enriched pathways in *Ncf1*^–/–^ PIL versus WT PIL renal neutrophils identified by Gene Set Enrichment Analysis (GSEA). Bars represent the Normalized Enrichment Score (NES). **B** Violin plots comparing expression levels of proinflammatory cytokines (*Il1b, Tnfa*), chemokines (*Ccl3, Ccl4, Cxcl2*), and chemokine receptors (*Cxcr4*) in WT and *Ncf1*^–/–^ neutrophils. **C** Violin plot of *Nlrp3* expression (top left) and box plot of AUCell enrichment scores for the NLRP3 inflammasome core pathway (top right) derived from scRNA-seq data. **D** RT-qPCR analysis of inflammasome-related genes (*Il1b, Nlrp3, Casp1*) in renal neutrophils: WT PIL (n = 4), *Ncf1*^–/–^ PIL (n = 5), data are normalized to β-actin. **E** Flow cytometric analysis of intracellular pro-IL-1β in renal neutrophils. Left: Quantification of geometric mean fluorescence intensity (Geo MFI) in WT PIL (n = 8) and *Ncf1*^−/−^ PIL (n = 7) mice. Right: Representative histograms showing pro-IL-1β fluorescence intensity. Data were pooled from two independent experiments and are presented median with IQR. Statistical Analysis: Significance was determined using the Wilcoxon rank-sum test for all panels (****p < 0.0001, ***p < 0.001, **p < 0.01, *p < 0.05, ns: not significant)

To elucidate the transcriptional dysregulation driving renal pathology in NOX2 deficient mice, we first evaluated the global transcriptomic profiles of renal neutrophils from WT and *Ncf1*^–/–^ mice with PIL via bulk-RNA sequencing on renal neutrophils from WT and *Ncf1*^–/–^ mice with PIL. Principal component analysis revealed a distinct segregation between the two genotypes (Figure S3A). Differential gene expression analysis disclosed upregulated proinflammatory mediators and pathway enrichment (Figure S3B, S3C). To further dissect transcriptomic profiles, we conducted scRNA-seq on renal neutrophils from WT and *Ncf1*^–/–^ mice with PIL. Pathway enrichment analysis revealed that *Ncf1*^–/–^ PIL neutrophils were significantly primed for inflammation, exhibiting strong enrichment of proinflammatory pathways compared to WT PIL controls (Fig. 3A). Consistent with this hyper-inflammatory state, *Ncf1*^–/–^ PIL neutrophils displayed a distinct transcriptional signature characterized by the significant upregulation of key cytokines (*Il1b, Tnfa*), chemokines (*Ccl3, Ccl4, Cxcl2*), and chemokine receptor (*Cxcr4*) (Fig. 3B). Given that NLRP3 inflammasome activation is the canonical pathway for IL-1β production, we hypothesized that this pathway is hyperactivated in NOX2-deficient neutrophils. To test this hypothesis, we analyzed a core NLRP3 inflammasome gene signature, including *Nlrp3, Pycard, Casp1, Il1b, Il18,* and *Gsdmd*, in neutrophils from the kidneys of WT and *Ncf1*^–/–^ PIL. Renal neutrophils from *Ncf1*^–/–^ PIL displayed significantly elevated expression of the NLRP3 inflammasome gene signature, whereas other innate sensors like NLRP12 and NOD2 were expressed at low levels and were not significantly changed (Fig. 3C). We further validated the relative mRNA expression levels of *Il1b*, *Nlrp3*, and *Casp1* by qPCR. When compared with WT PIL, *Ncf1⁻/⁻* PIL renal neutrophils exhibited a 2.15-fold increase in *Il1b*, a 1.70-fold increase in *Casp1*, and a 1.87-fold increase in *Nlrp3* (Fig. 3D). To confirm the elevated production of IL-1β at the protein level, we analyzed kidney neutrophils using intracellular flow cytometry. We observed that *Ncf1*^−/−^ PIL mice exhibited a significantly augmented geometric mean fluorescence intensity for pro-IL-1β compared to WT PIL controls (Fig. 3E). To further characterize the inflammatory milieu within the kidneys, we assessed the spatial distribution and expression levels of NLRP3. Immunofluorescence staining revealed extensive colocalization of NLRP3 with Ly6G^+^ neutrophils specifically within the glomeruli of *Ncf1*^−/−^ lupus mice. In contrast, this pattern of glomerular expression was markedly reduced in WT PIL (Figure S4). Corroborating these histological observations, immunoblot analysis of the kidney cortex confirmed that total NLRP3 protein levels were significantly elevated in *Ncf1*^−/−^ lupus mice compared to WT PIL (Figure S5). Given that neutrophil extracellular trap (NET) formation is a critical driver of SLE and LN pathogenesis [19], and that NETosis can occur via ROS-dependent or -independent mechanisms [20], we investigated whether NOX2 deficiency affects the capacity of neutrophils to undergo NETosis. Whereas stimulation with PMA elicited robust NET formation in WT neutrophils, this response was completely abrogated in *Ncf1*^–/–^ neutrophils (Figure S6A and S6B). Furthermore, because NETosis can also occur via NOX2-independent mechanisms, we sought to determine whether spontaneous or NOX2-independent NETosis occurs in vivo. We quantified the levels of citrullinated histone H3 (CitH3), an established marker of NETosis. Notably, CitH3 levels showed no significant differences between the *Ncf1*^*−/−*^ and WT PIL mice (Figure S6C). Thus, we found no evidence that NOX2 deficiency increases renal CitH3 accumulation. Collectively, these findings indicate that NOX2 deficiency attenuates NET formation and simultaneously fosters a hyper-inflammatory phenotype via increased NLRP3 inflammasome activation in renal neutrophils.

### Identification of a neutrophil subset characterized by enhanced NF-κB signaling and prolonged survival in the NOX2 deficient mice with nephritis

**Fig. 4:**
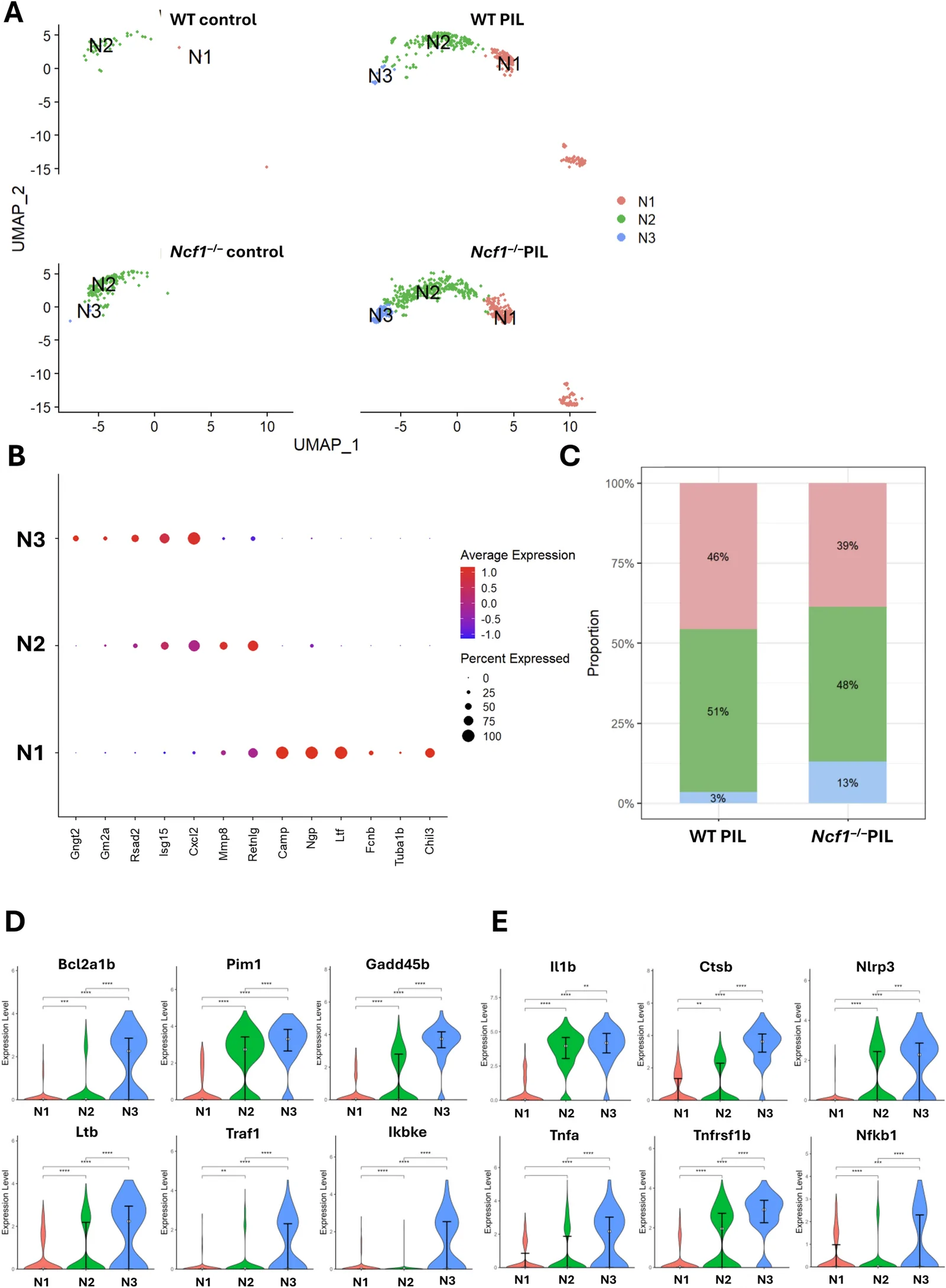
NOX2 deficiency promotes the expansion of a hyper-inflammatory, anti-apoptotic neutrophil subset.** A** Uniform Manifold Approximation and Projection (UMAP) visualization of integrated renal neutrophil subpopulations (N1, N2, N3) segregated by experimental condition: WT control, WT PIL, *Ncf1*^–/–^ control, and *Ncf1*^–/–^ PIL. **B** Dot plot displaying the scaled average expression and percentage of cells expressing top marker genes defining clusters N1–N3. **C** Stacked bar charts quantifying the frequency of neutrophil clusters (N1–N3) within the total neutrophil compartment of WTP and KOP mice. **D** Violin plots assessing the expression levels of gene modules associated with NF-κB signaling (*Traf1, Ikbke, Ltb*), anti-apoptosis/survival (*Bcl2a1b, Pim1, Gadd45b*) **E** Violin plots quantifying expression of NLRP3 inflammasome pathway components (*Il1b, Nlrp3, Ctsb, Tnf, Tnfrsf1b, Nfkb1*). Data are represented as median with IQR. ***p < 0.001, ****p < 0.0001 (Kruskal–Wallis test)

To elucidate the transcriptional heterogeneity of renal neutrophils during tissue injury, we performed unbiased sub-clustering analysis on the integrated scRNA-seq dataset. We interpreted these subsets using the developmental framework established by Xie et al., which delineates a continuum from proliferative precursors to terminally differentiated neutrophils possessing an IFN signature [21]. Our analysis resolved three transcriptionally distinct states (N1–N3) (Fig. 4A). Cluster N1 exhibited a phenotype consistent with early-stage neutrophils, defined by high expression of granule proteins (*Camp, Ngp, Ltf*), whereas N2 was enriched for maturation and migration markers (*Mmp8, Retnlg*). In contrast, the N3 cluster displayed a robust inflammatory profile analogous to the terminal subset characterized by elevated expression of IFN-stimulated genes (*Isg15, Rsad2*) and chemokines (*Cxcl2*) (Fig. 4B). Notably, while N3 cells were rare in WT PIL (3%), this population underwent a marked four-fold increase (13%) in the kidneys of *Ncf1*^–/–^ PIL mice (Fig. 4C). Specifically, pseudotime trajectory analysis revealed a sequential maturation continuum from N1 to N3 neutrophil subclusters (Figure S7A and S7B). To determine if this hyper-inflammatory state was intrinsic to the expanded N3 subset, we analyzed cluster-specific signatures. Indeed, N3 neutrophils exhibited a distinct anti-apoptotic program characterized by the significant upregulation of *Bcl2a1b, Pim1*, and *Gadd45b* compared to N1 and N2 clusters (Fig. 4D). To evaluate the capacity for IL-1β secretion, we analyzed the transcriptomic profiles of the three clusters. The N3 population exhibited significant upregulation of upstream NF-κB priming signals (*Tnfa, Tnfrsf1b, Nfkb1*) and essential components of the NLRP3 pathway (*Il1b, Nlrp3, Ctsb*) compared with the remaining clusters (Fig. 4E). As cells advanced along pseudotime, they robustly upregulated hallmark inflammatory cascades (Figure S7C). Collectively, these data suggest that NOX2 deficiency potentiates renal damage by promoting the survival and accumulation of a highly inflammatory, NLRP3-active neutrophil subset.

### IL-1 receptor blockade attenuates renal pathology in NOX2-deficient mice

**Fig. 5:**
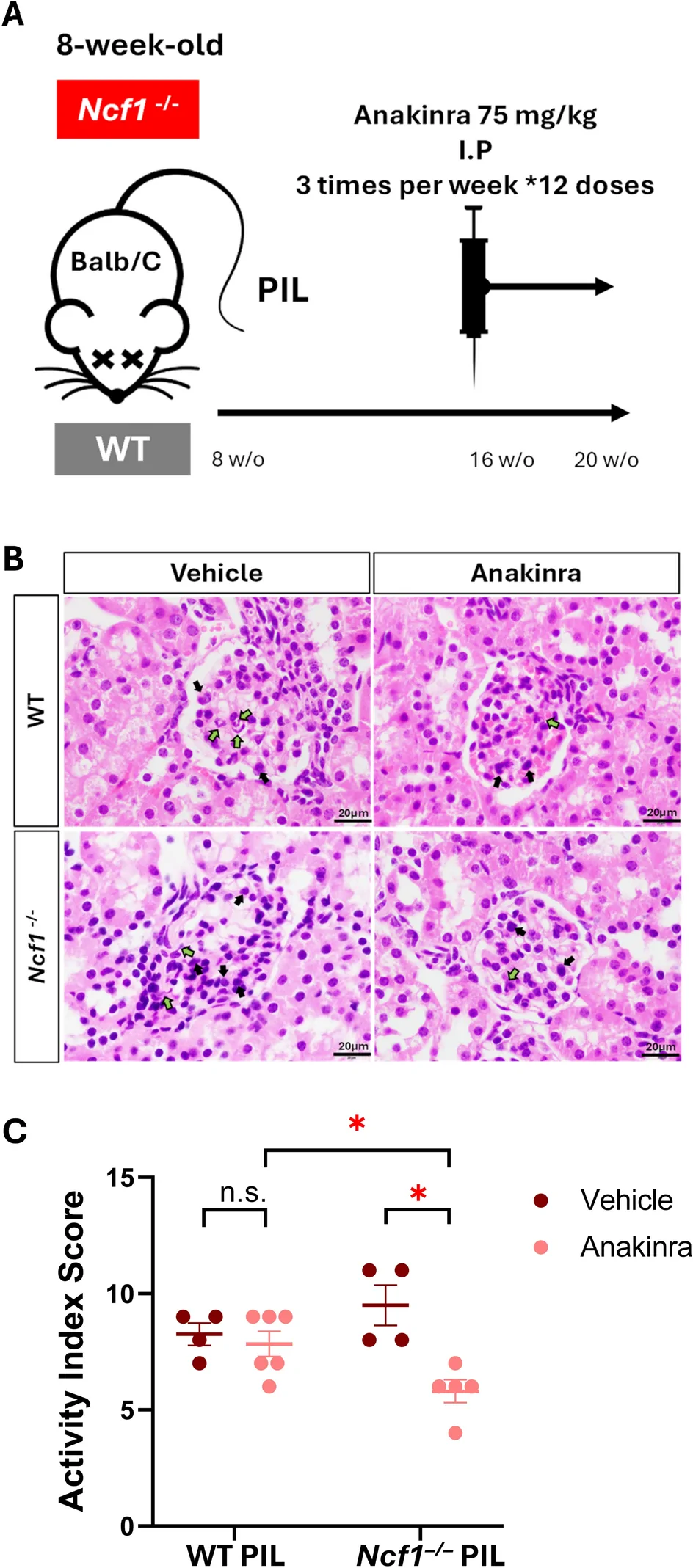
IL-1 blockade mitigates renal disease activity in NOX2-deficient mice with PIL.** A** Experimental schema. Female *Ncf1*^–/–^ BALB/c mice (8 weeks old) were injected with pristane (0.5 ml, I.P.). Starting at 16-week-old (8 weeks post-induction), mice were treated with the IL-1 receptor antagonist anakinra (75 mg/kg i.p., 3 times/week for 12 doses) until 20 weeks of age. **B** Representative H&E-stained kidney sections showing glomerular morphology in WT PIL and *Ncf1*^–/–^ PIL mice following anakinra treatment. Scale bars: 20 µm. Black arrows indicate hypertrophied mesangial cells; green arrows indicate neutrophils infiltration. Scale bar = 20 μm. **C** Quantification of renal disease severity using the activity index score across the experimental groups: WT PIL vehicle (n = 4), WT PIL anakinra (n = 6), *Ncf1*^–/–^ PIL vehicle (n = 4), and *Ncf1*^–/–^ PIL anakinra (n = 5). Data are expressed as the median [IQR]. Statistical significance was determined using the Wilcoxon rank-sum test (*p < 0.05)

Given that NOX2 deficiency elevates IL-1β production and exacerbates glomerular injury, we next investigated whether enhanced IL-1 signaling directly drives this severe lupus nephritis phenotype. Following disease induction, both the WT and *Ncf1*^–/–^ PIL mice received intraperitoneal injections of anakinra, an IL-1 receptor antagonist**,** to abrogate IL-1 signaling during the established phase of the disease (Fig. 5A). Following the 4-week treatment regimen, we assessed the extent of glomerular inflammation. Histopathological evaluation of renal tissue revealed visibly reduced inflammation in *Ncf1*^–/–^ PIL group compared to the WT PIL group after anakinra treatment (Fig. 5B). Consistent with our histological findings, anakinra treatment significantly attenuated renal activity index scores in *Ncf1*^*–/–*^ PIL mice compared with vehicle-treated controls (*p* < 0.05, Fig. 5C). In contrast, no significant difference was observed between anakinra-treated and vehicle-treated WT PIL mice. Notably, following anakinra treatment, the renal activity index scores were significantly lower in *Ncf1*^*–/–*^ PIL mice than in identically treated WT PIL mice (*p* < 0.05, Fig. 5C). Anakinra antagonizes both IL-1α and IL-1β signaling. To determine whether IL-1α contributes to the therapeutic efficacy in our model, we quantified *Il1a* transcripts in renal neutrophils and showed that *Il1a* expression was negligible in both WT and *Ncf1*^−/−^ mice (Figure S8). Furthermore, IL-1α protein levels in kidney homogenates from these mice were barely detectable (data not shown). These results indicate that the therapeutic benefits of anakinra are predominantly mediated by abrogating the upregulated IL-1β pathway.

## Discussion

In this study, we demonstrated that NOX2 deficiency paradoxically exacerbates LN in a pristane-induced murine model. Rather than relying on classical NETosis, this fulminant autoimmune phenotype is driven by the transcriptional reprogramming of renal neutrophils. Specifically, single-cell transcriptomics revealed that the absence of NOX2 triggers a compensatory hyperactivation of the NLRP3/IL-1β axis, promoting the aberrant expansion of a pathogenic neutrophil subset defined by a robust interferon signature. Crucially, therapeutically validating this mechanism with targeted IL-1 receptor blockade effectively attenuated renal disease activity.

NOX2-derived ROS are indispensable to the microbiocidal arsenal of phagocytic leukocytes [22]. Functional impairment of the NOX2 complex abrogates this respiratory burst, forming the molecular etiology of CGD [23]. Paradoxically, ROS deficiency not only confers immunodeficiency but also actively drives chronic hyperinflammation and autoimmune dysregulation [24–26]. Clinically, this paradox is underscored by the high frequency of autoimmune complications in patients with CGD [27, 28]. At the genomic level, polymorphisms within *NCF1* serve as profound susceptibility loci for autoimmune conditions; these variants confer markedly elevated risk, particularly for the development of SLE [29, 30]. In agreement with these genetic and clinical observations, defective NOX2-dependent ROS production in granulocytes is intrinsically linked to lupus pathogenesis. Specifically, attenuated neutrophil ROS generation correlates inversely with systemic inflammatory markers including anti-dsDNA autoantibody titers and glomerulonephritis [7, 29–31]. Our findings robustly substantiate the ROS deficiency hypothesis in SLE. Furthermore, these data align with clinical observations linking impaired granulocytic oxidative bursts to exacerbated disease severity and progressive organ damage [32, 33].

Our previous studies using the K/BxN arthritis model established how phagocyte-derived ROS limits joint inflammation. Huang et al. demonstrated that NOX2-deficient (*Ncf1*^*–/–*^) mice develop severe arthritis with elevated IL-1β, as ROS restricts cathepsin B and caspase-1-mediated IL-1β processing [8]. This IL-1β-rich environment subsequently drives innate lymphoid cell plasticity, which is reversible via IL-1 blockade [34]. Furthermore, Liao et al. recently identified that NOX2 deficiency fundamentally alters the articular neutrophil phenotype. However, prior investigations have not delineated the interplay between the NLRP3 inflammasome and NETosis specifically within the context of NOX2-deficient neutrophils. Our findings address this knowledge gap, revealing a distinct pathogenic mechanism where severe nephritis can develop independently of classical NETosis, propelled instead by a hyperactive NLRP3 cascade. This paradigm closely aligns with recent observations in murine ANCA-associated glomerulonephritis. In that distinct disease model, NOX2 deficiency also exacerbated renal injury via enhanced inflammasome activation, and therapeutic intervention with the IL-1 receptor antagonist anakinra significantly attenuated glomerular leukocytes infiltration [35]. Taken together, these parallel observations suggest that while NOX2 deficiency broadly amplifies autoimmune susceptibility, the dominant inflammatory pathways and cellular effectors are highly adapting to specific tissue microenvironments and diverse disease etiologies.

Beyond its established pathogenic involvement in lupus and inflammatory arthritis, NOX2 exhibits pleiotropic effects that provide protection against acute tissue inflammation and metabolic dysfunction [36]. For example, in a murine model of zymosan-induced lung inflammation, NOX2-deficient neutrophils amplify the IL-1β/G-CSF axis and promote severe pulmonary inflammation [37]. Systemically, NOX2 also confers significant metabolic benefits; mice lacking NOX2 exhibit exacerbated diet-induced obesity, worsened whole-body insulin resistance, and accelerated aortic plaque formation [38, 39]. Importantly, as highlighted by recent studies, this immunomodulatory capacity establishes NOX2 as a negative regulator of inflammatory signaling rather than merely a ROS producer [40, 41]. Mechanistically, this relies on its control over intracellular signaling, organelle homeostasis, and cellular bioenergetics. First, NOX2 actively suppresses pro-inflammatory transcription by sequestering thioredoxin-1 (TRX1) at the cell membrane, which maintains NF-κB in an oxidized and inactive state; conversely, NOX2 deficiency hyperactivates NF-κB to drive exaggerated inflammation [42]. Second, NOX2 heavily regulates organelle microenvironments. In dendritic cells, NOX2-derived superoxide neutralizes phagosomal protons to prevent excessive acidification. This precise redox and pH regulation protects engulfed antigens from complete enzymatic destruction, a step essential for successful cross-presentation to T cells [43, 44]. Finally, mitochondrial-localized NOX2 was reported to help coordinate redox signaling with mitochondrial oxidative phosphorylation, thereby maintaining bioenergetic flexibility in hematopoietic stem and progenitor cells during stress responses [41]. While WT cells can mount a respiratory adaptive response to oxidative stress or danger signals, NOX2-deficient cells lose this adaptability. Consequently, they undergo a forced metabolic shift toward anaerobic glycolysis, leaving them in a state of chronic oxidative stress. Collectively, these multifaceted functions underscore the paradoxical but critical role of NOX2 as a master modulator of innate immune regulation [40].

Neutrophils are central effectors in the pathogenesis of LN, driving localized inflammation, tissue damage, and fibrotic remodeling; consequently, they serve as cardinal histological markers of active disease [13]. While established models indicate that glomerular neutrophil recruitment is canonically initiated by FcγRIIA-mediated capture of immune complexes and sustained by Mac-1-dependent adhesion [24], our findings reveal a crucial, cell-intrinsic mechanism that amplifies this process. We observed a marked upregulation of chemokine and chemokine receptor transcripts in NOX2-deficient neutrophils. Because WT and *Ncf1*^–/–^ mice exhibited comparable levels of immune complex deposition (Figure S1), this robust chemotactic signature demonstrates that NOX2 deficiency confers an intrinsically hyper-recruitable phenotype to neutrophils. NOX2-sufficient neutrophils canonically exacerbate tissue injury through the release of NETs. Specifically, previous work demonstrates that NET-derived CitH3 engages mesangial TLR4 to drive cellular proliferation and fibrosis [45]. Classical NETosis relies heavily on ROS derived from NOX2; consequently, NOX2 deficiency typically abrogates this pathway. Unexpectedly, our results demonstrated that renal CitH3 levels did not differ between WT and *Ncf1*^–/–^ PIL mice. These data indicate that NOX2 deficiency does not alter renal CitH3 accumulation in this model. Nevertheless, this observation does not preclude the occurrence of spontaneous or NOX2-independent NETosis. Confirming the presence of these alternative mechanisms requires further NET-specific evaluations, such as quantifying MPO-DNA complexes or assessing the colocalization of extracellular CitH3, MPO, and DNA. Crucially, our transcriptomic data maps this hyperactive phenotype to a specific developmental trajectory, progressing from Cluster N1 to Cluster N3. We observed that NOX2 deficiency not only drives this continuum but also facilitates the prolonged survival and pathogenic accumulation of the N3 subset. This highly inflammatory, NLRP3-active neutrophil population is uniquely characterized by the upregulation of hallmark inflammatory cascades and key NLRP3 pathway components. Strikingly, the transcriptomic signature of this murine N3 cluster closely mirrors that of pathogenic low-density granulocytes previously identified in human SLE [46].

Given that macrophages also express NLRP3 and IL-1β, we compared the macrophage and neutrophil compartments in our scRNA-seq dataset in this study. While *Nlrp3* expression was moderately higher in macrophage clusters from *Ncf1*^*−/−*^ PIL mice when compared with those from WT PIL mice (Figure S9A), *Il1b* transcript levels were not different between the two groups (Figure S9B). On the contrary, neutrophil clusters from *Ncf1*^*−/−*^ PIL mice displayed significant and concomitant upregulation of both *Nlrp3* and *Il1b* relative to those from WT PIL mice (Fig. 3B, 3 C). Following pristane induction, the neutrophil frequencies increased in both WT and *Ncf1*^*−/−*^ mouse groups (Fig. 2B), whereas KRM frequencies decreased in both groups (Fig. 2C). We therefore proceeded to characterize the differences in kidney neutrophils from *Ncf1*^*−/−*^ PIL mice in comparison with those from WT mice to reveal their activity in renal inflammation.

The precise mechanisms governing how NOX2 deficiency drives NLRP3 activation in neutrophils remain incompletely understood. Recent evidence by Monjarret et al. [12] demonstrates that NOX2 deficiency in macrophages induces profound mitochondrial damage. This defect elevates mitochondrial ROS (mtROS), which paradoxically amplifies NLRP3 activation. A critical downstream cascade linking this excessive mtROS to NLRP3 assembly involves the TRX1 and thioredoxin-interacting protein (TXNIP) regulatory axis. Under homeostatic conditions, TRX1 binds and represses TXNIP. Conversely, elevated mtROS triggers their dissociation, enabling TXNIP to actively promote NLRP3 inflammasome assembly [47]. In line with this mechanism, our single-cell transcriptomic dataset revealed a significant downregulation of *Trx1* alongside a concurrent upregulation of *Txnip* in *Ncf1*^*−/−*^ renal neutrophils compared to wild-type controls (Figure S10). Collectively, our transcriptomic profiles, contextualized by current literature, suggest that the absence of NOX2 in *Ncf1*-deficient neutrophils creates a permissive environment for NLRP3 activation connected to an mtROS-driven TRX1/TXNIP signaling axis. While these findings offer a compelling mechanistic rationale, the transcriptomic shifts may not fully capture posttranslational dissociation events. Consequently, targeted in vitro cell-based assays and in vivo animal investigations are warranted to definitively validate this signaling cascade at the protein level.

The pathogenic role of IL-1β in SLE and LN remains controversial. Although intrarenal IL-1β is robustly elevated in lupus-prone mice [48], therapeutic blockade of this pathway has previously failed to ameliorate established nephritis [49, 50]. Conversely, IL-1 inhibition has demonstrated therapeutic potential in other renal pathologies, such as AA amyloidosis-associated kidney disease [51] and chronic kidney disease [52, 53]. Notably, previous studies demonstrating the failure of anti-IL-1 therapy utilized NOX2-sufficient mouse models without a targeted genetic stratification strategy. This phenotypic complexity is mirrored in human cohorts, where the correlation between *IL1B* single nucleotide polymorphisms and disease susceptibility exhibits significant population-dependent variability [54–56]. Collectively, these discordant observations suggest that IL-1β may not be a universal driver of LN but rather a context-dependent effector whose pathogenic potential is unmasked within specific genetic landscapes that prime the inflammasome axis. In support of this, a study of mononuclear phagocytes derived from patients with CGD demonstrated that NOX2 deficiency precipitates caspase-1 activation and subsequent IL-1β secretion [57]. Consistent with these reports, our data showed that IL-1β was significantly less abundant in WT NOX2-sufficient PIL mice compared to NOX2-deficient PIL mice. Consequently, IL-1 blockade failed to reduce renal inflammation in the NOX2-sufficient group. In contrast, NOX2 deficiency in murine lupus promoted robust IL-1β accumulation within inflamed renal tissues, rendering these mice highly responsive to targeted IL-1 inhibition.

While current international guidelines [58, 59] emphasize early combination therapies, ranging from glucocorticoids to novel B-cell depleting agents, up to 60% of patients fail to achieve complete renal remission, leaving them at high risk for end-stage kidney disease. This persistent therapeutic gap suggests that targeting adaptive immunity alone may be insufficient for patient subsets driven by innate inflammatory dysregulation. Although therapeutic IL-1 blockade currently remains outside the standard of care for SLE and LN and has been deployed only in isolated or refractory clinical scenarios [60, 61], our data strongly argue that prior clinical disappointments likely reflect inadequate patient stratification rather than fundamental target invalidity. In this study, we identify IL-1β as the key pathogenic effector of renal injury driven by NOX2 deficiency-mediated NLRP3 hyperactivation. Therapeutically, IL-1 receptor blockade with anakinra robustly ameliorated established lupus nephritis and reversed histological damage in *Ncf1*^*–/–*^ PIL mice. Although N2 neutrophils predominate in WT PIL mice and actively transcribe *Il1b*, anakinra treatment lacks efficacy in this cohort. This disparity likely reflects the post-translational requirement for NLRP3-mediated IL-1β activation. Our transcriptomic data reveal that despite robust *Il1b* transcription, N2 neutrophils express less *Nlrp3* compared to the N3 subset. This drives significantly increased renal IL-1β abundance (Fig. 1H) and increased intracellular pro-IL-1β staining in renal neutrophils (Fig. 3E), rendering *Ncf1*-deficient pathogenesis highly dependent on the IL-1β axis. The lack of N3-driven IL-1β maturation in WT mice thus provides a mechanistic rationale for their unresponsiveness to anakinra. Together, our findings delineate a distinct pathophysiological mechanism and expose aberrant innate IL-1β production as a clear therapeutic vulnerability. These results provide a strong rationale for revisiting IL-1 inhibition as a precision therapy for lupus nephritis patients stratified by genetic variants, such as *NCF1* polymorphisms.

Our findings provide vital mechanistic insights into how NOX2-deficient neutrophils exacerbate the immune pathogenesis of LN, identifying the IL-1β pathway as a promising therapeutic target. However, we acknowledge certain limitations within this study. First, the N3 neutrophil subset was identified exclusively via single-cell transcriptomic profiling. The current absence of unique cell-surface markers, compounded by the fragile nature of primary renal neutrophils preventing their ex vivo expansion, precluded fluorescence-activated cell sorting and subsequent adoptive transfer to definitively isolate their in vivo effects from other immune cells. Second, our reliance on a systemic *Ncf1* knockout model limits the ability to definitively exclude pathogenic contributions from NOX2 deficiency in other immune compartments, as *Ncf1* is broadly expressed. Future investigations employing cell-type-specific Cre-LoxP conditional knockout systems are required to unravel these intercellular dynamics and isolate the precise pathogenic role of NOX2-deficient neutrophils. Despite these experimental constraints, our strategy to target the downstream effector of NLRP3 using the IL-1 receptor antagonist anakinra provides a robust proof of concept for in vivo therapeutic intervention. By prioritizing a widely accessible, FDA-approved biologic over direct but currently unapproved NLRP3 inhibitors, this approach maximizes the potential for immediate clinical translation. Consequently, our data established a strong rationale for future clinical trials to evaluate anakinra as a repurposed precision treatment for patients with LN, specifically those harboring a NOX2-deficient genetic background.

## Conclusions

In conclusion, this study elucidates a novel immunoregulatory mechanism wherein NOX2-derived ROS suppresses NLRP3 inflammasome activation in renal neutrophils. We provide evidence that NOX2 deficiency drives a specific, IL-1β-dependent pathway of glomerular injury. These findings not only clarify the pathogenic function of *NCF1* variants but also highlight the potential of genotype-guided IL-1β blockade as a precision therapeutic strategy for SLE.

## Supplementary Information

Supplementary material 1

Supplementary material 2

## Data Availability

The datasets generated and analyzed during the current study are available within the article and its supplementary material files. Any further inquiries should be directed to the corresponding author upon reasonable request.

## Abbreviations

ANCA: Anti-neutrophil cytoplasmic antibodies
Casp: Caspases
CCL: CC chemokine ligand
CCR: CC chemokine receptor
CD: Cluster of differentiation
CGD: Chronic granulomatous disease
CitH3: Citrullinated histone H3
CXCL: CXC motif chemokine ligand
FDR: False discovery rate
GSDMD: Gasdermin D
GSEA: Gene set enrichment analysis
H&E: Hematoxylin and eosin
IFN: Interferon
IL: Interleukin
LN: Lupus nephritis
MFI: Mean fluorescence intensity
MPO: Myeloperoxidase
Ncf1: Neutrophil Cytosolic Factor 1
NETs: Neutrophil Extracellular Traps
NF-κB: Nuclear Factor Kappa B
NLRP3: Nucleotide-binding oligomerization domain leucine-rich repeat and pyrin domain containing 3
NOX2: NADPH oxidase 2
PD-L1: Programmed death-ligand 1
PMA: Phorbol 12-myristate 13-acetate
PYCARD: PYD and CARD Domain Containing
ROS: Reactive oxygen species
TNF-α: Tumor necrosis factor-α
PIL: Pristane-induced murine model of lupus
scRNA-seq: Single-cell RNA sequencing
SLE: Systemic lupus erythematosus
STAT: Signal transducer and activator of transcription
UMAP: Uniform manifold approximation and projection
WT: Wild type
TRX1: Thioredoxin-1
TXNIP: Thioredoxin-interacting protein
