# Supplementary material for: NOX2 deficiency exacerbates lupus nephritis through activation of NLRP3 inflammasome in neutrophils
Source: J Biomed Sci. 2026 Aug 19;33:84. doi: 10.1186/s12929-026-01287-2 (PMC13491832; doi:10.1186/s12929-026-01287-2)
Supplement: Supplementary file 1 [file 12929_2026_1287_MOESM1_ESM.pdf]

Supplementary Figures

Supplementary Figure S1

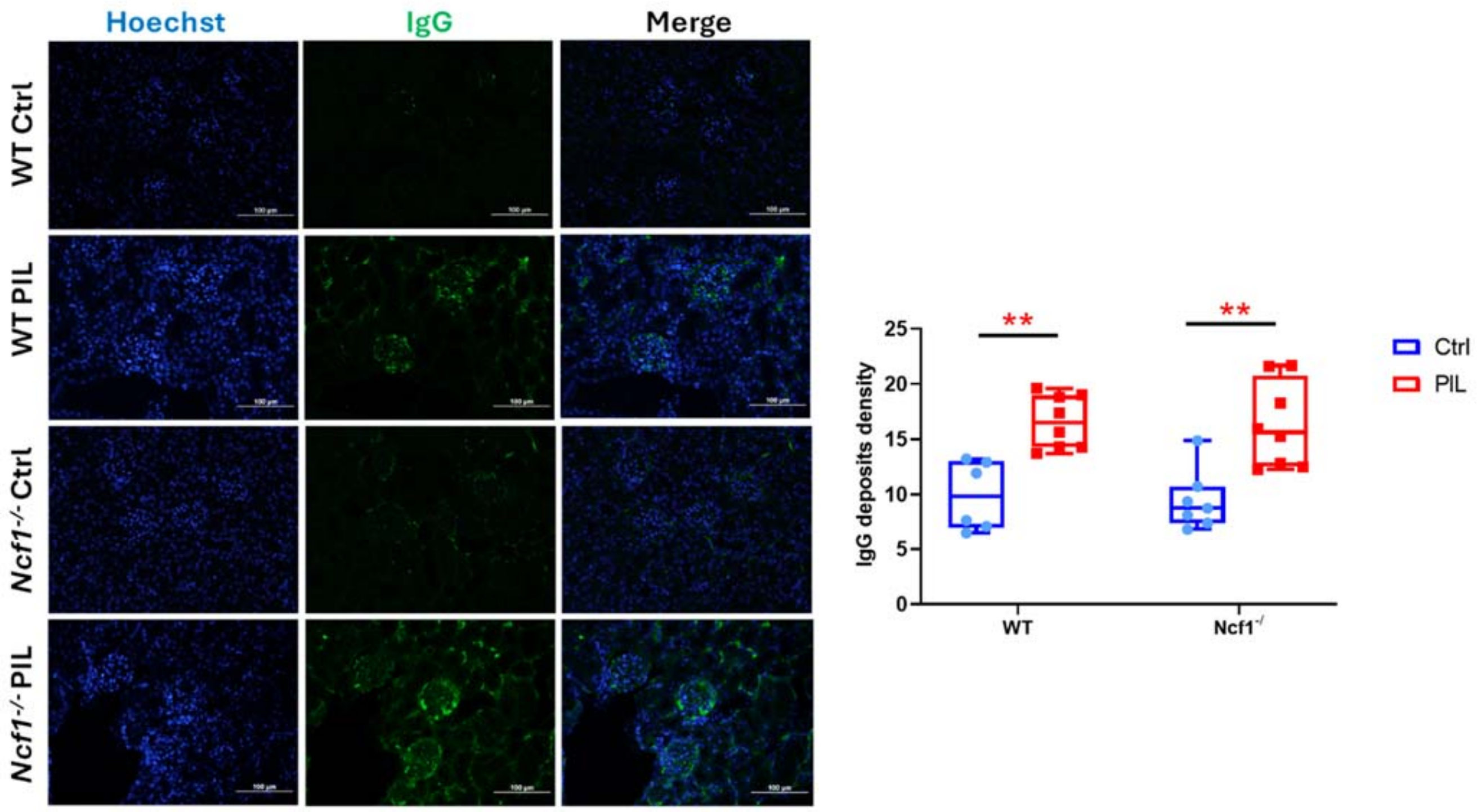

Figure S1. Glomerular IgG immune complex deposition is increased in PIL-induced mice.

(A) Representative immunofluorescence images of kidney sections from wild-type (WT) and *Ncf1*$^{-/-}$ mice. Sections were stained for IgG (green) to visualize immune complex deposits and Hoechst (blue) for nuclear counterstaining. Images show comparisons between controls and pristane-induced lupus (PIL) groups. Scale bar: 100 µm. **(B)** Quantification of glomerular IgG deposit density across four experimental groups: WT control (n=6), WT PIL (n=8), *Ncf1*$^{-/-}$ control (n=7), and *Ncf1*$^{-/-}$ PIL(n=8). Box plots represent the density of IgG deposits, showing a significant increase in both WT and *Ncf1*$^{-/-}$ PIL groups compared to their respective controls. Data are expressed as median with interquartile range. **$p < 0.01$ vs. corresponding controls.

Supplementary Figure S2

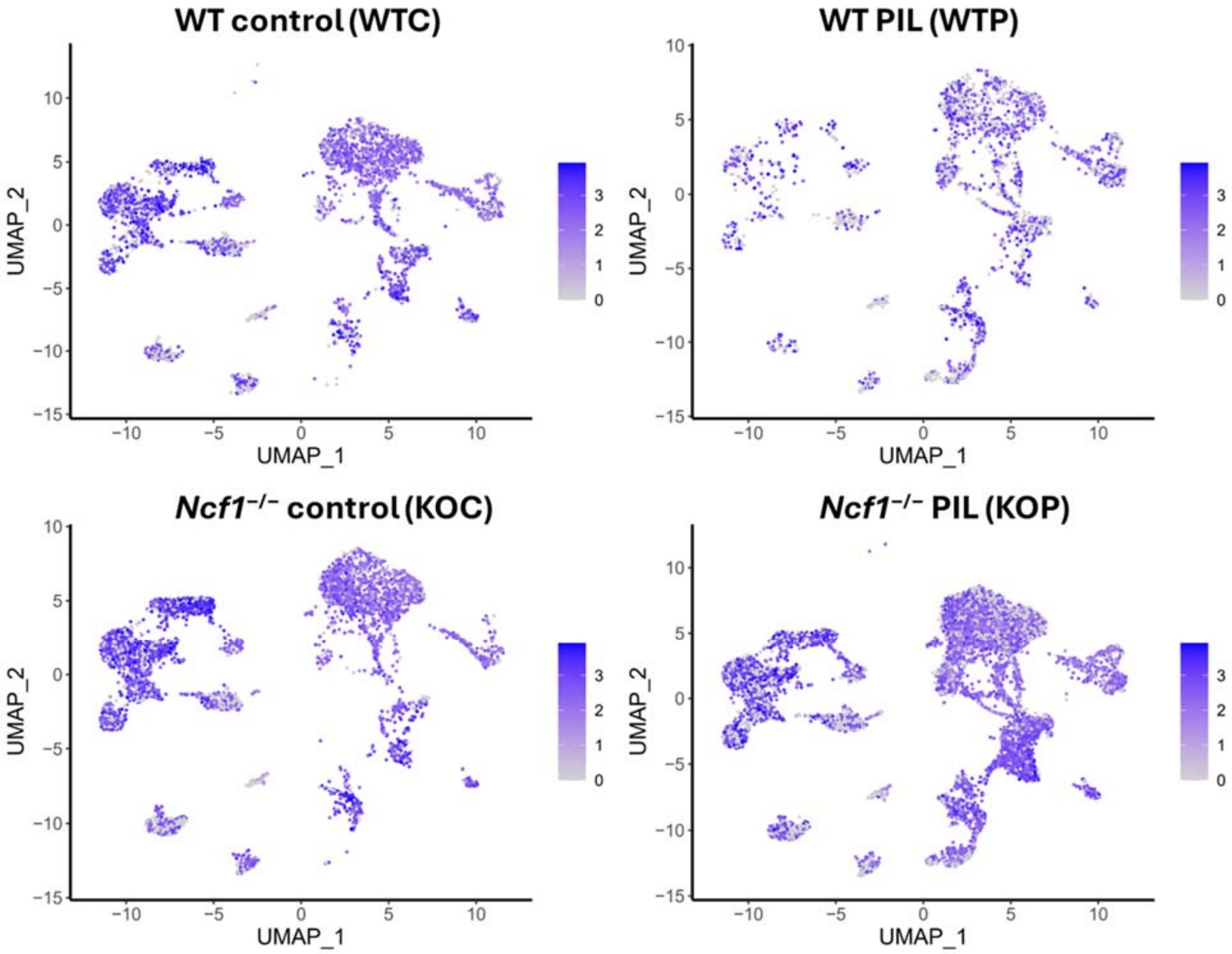

Figure S2. Validation of CD45+ leukocyte clusters from kidney immune cell scRNA-seq datasets. Uniform Manifold Approximation and Projection feature plots displaying the single-cell transcriptomic landscape. Cells are colored by the normalized expression level of the pan-leukocyte marker *Ptprc* (CD45), with dark blue indicating high expression.

Supplementary Figure S3

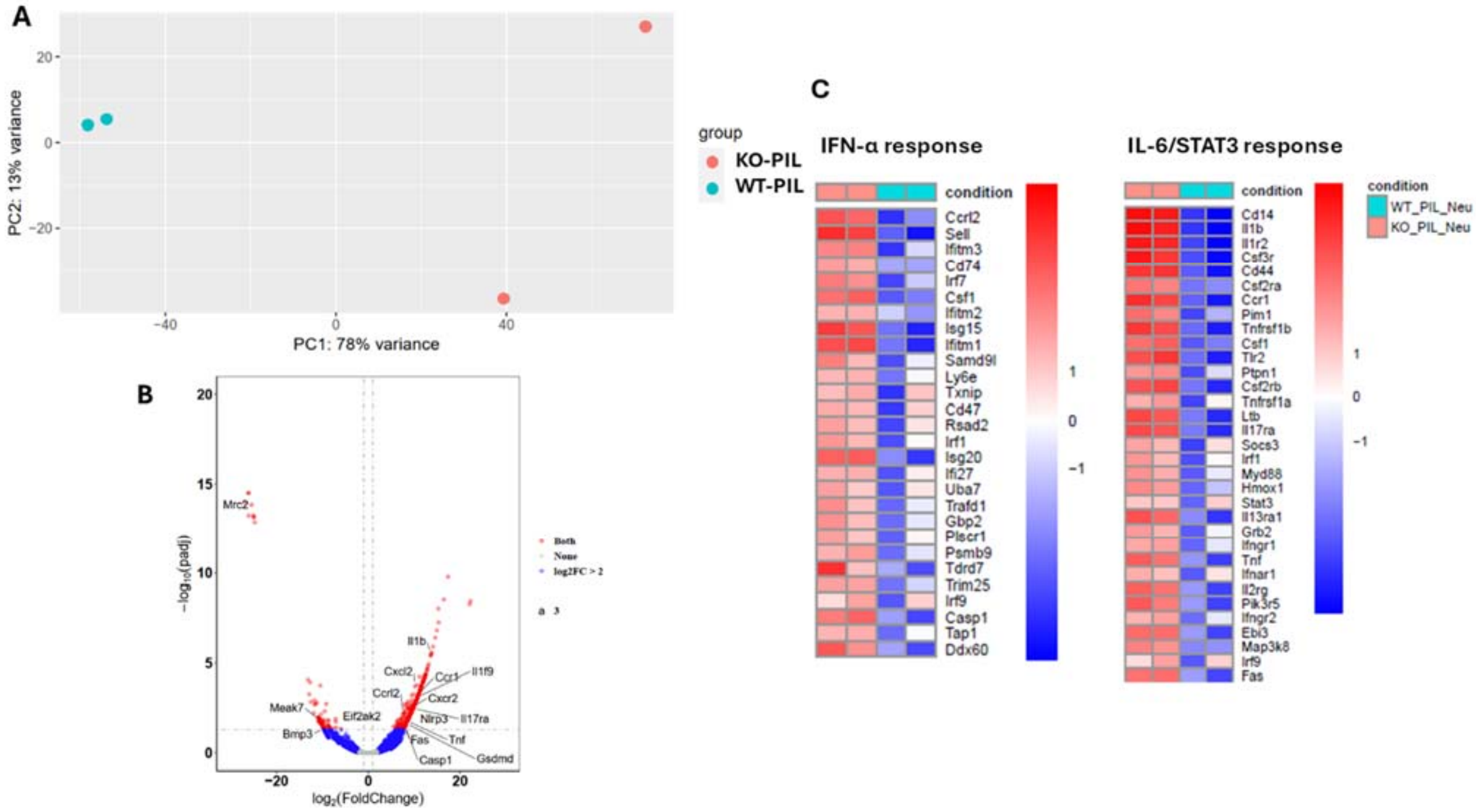

Figure S3. Transcriptional profiles of WT and *Ncf1*$^{-/-}$ renal neutrophils during pristane-induced lupus. (A) Principal component analysis of bulk RNA-seq data from renal neutrophils isolated from WT (blue, n= [2]) and *Ncf1*$^{-/-}$ (red, n= [2]) mice after pristane induction, demonstrating clear transcriptomic segregation by genotype. (B) Volcano plot displaying differentially expressed genes between *Ncf1*$^{-/-}$ and WT renal neutrophils. Significantly upregulated and downregulated genes (adjusted $p< 0.05$ and log2FC > 2) are highlighted in red and blue, respectively. Key proinflammatory genes are annotated. (C) Heat maps show up-regulated genes of IFN-α response and IL-6/STAT3 signaling from *Ncf1*$^{-/-}$ renal neutrophils.

Supplementary Figure S4

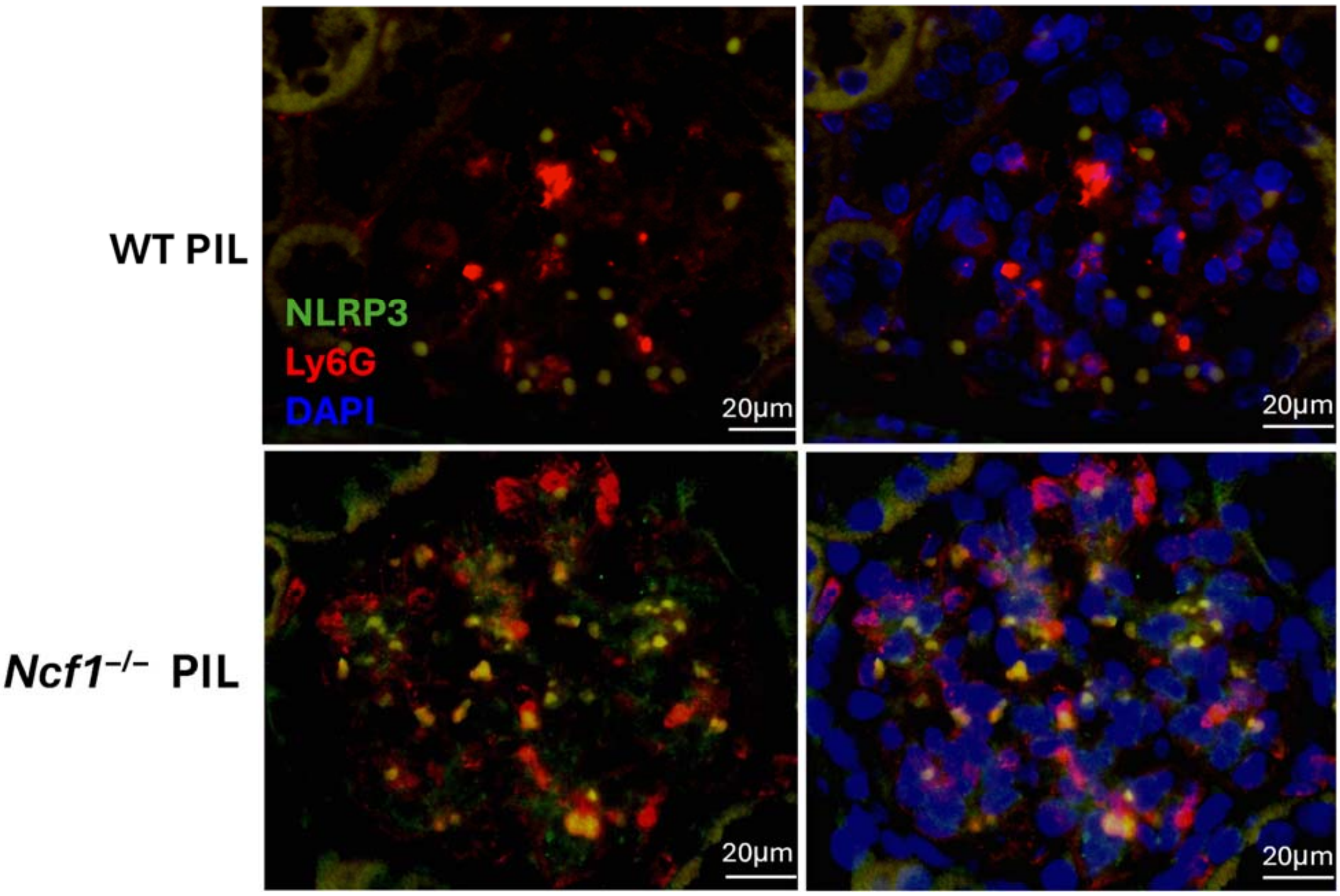

Figure S4. Immunofluorescence analysis of NLRP3 and Ly6G expression in the glomerulus. Representative images of kidney sections from WT and *Ncf1*$^{-/-}$ mice in the PIL group. Sections were stained for NLRP3 (green), the neutrophil marker Ly6G (red), and DAPI (blue). The top row (WT PIL) and bottom row (*Ncf1*$^{-/-}$ PIL) show the localization of neutrophils within the glomerular tuft. Scale bars: 20 μm.

Supplementary Figure S5

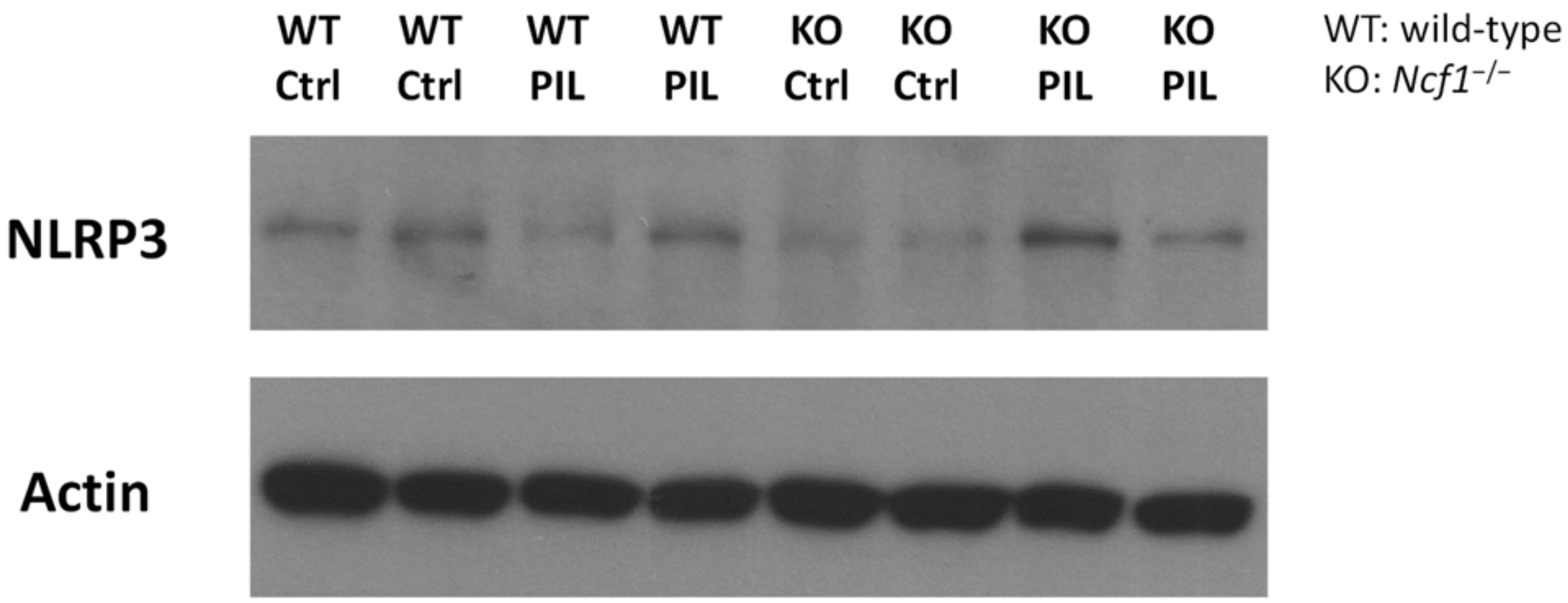

Figure S5. NLRP3 protein expression in the kidney cortex. Representative Western blot images show the protein levels of NLRP3 in kidney cortex tissue lysates from WT and *Ncf1*$^{-/-}$ mice. Actin served as an internal loading control to ensure equal protein loading. The experiment was repeated two times with similar results.

Supplementary Figure S6

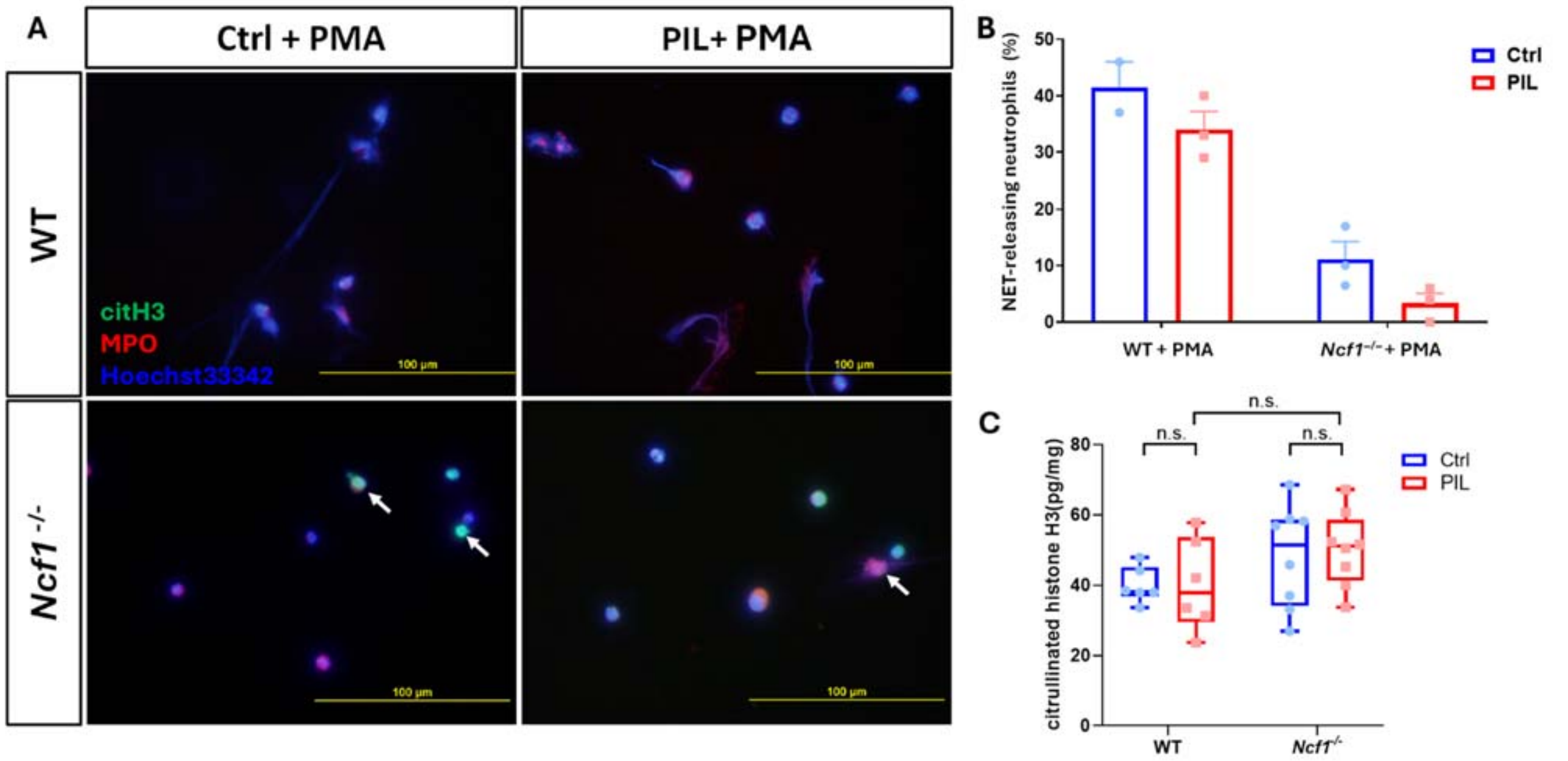

Figure S6. Impaired NETosis in NOX2-deficient neutrophils. (A) Representative immunofluorescence images of bone marrow-derived neutrophils from WT and *Ncf1*$^{-/-}$ mice following stimulation with PMA (100 nM). Cells were stained for the NETosis marker citH3 (green), Myeloperoxidase (MPO, red), and DNA (Hoechst 33342, blue). While WT neutrophils exhibited extensive chromatin decondensation and NET release, *Ncf1*$^{-/-}$ neutrophils largely retained intact nuclei. White arrows indicate neutrophils with condensed nuclei that failed to undergo NETosis. Scale bars: 100 µm. (B) Percentage of NET-releasing neutrophils across the four experimental groups: WT control (n=2), WT PIL (n=3), *Ncf1*$^{-/-}$ control (n=3), and *Ncf1*$^{-/-}$ PIL(n=3). Data are presented as mean ± SEM. **(C)** Quantification of the citrullinated histone H3 in kidney homogenates from four experimental groups: WT control (n=5), WT PIL (n=6), *Ncf1*$^{-/-}$ control (n=8), and *Ncf1*$^{-/-}$ PIL(n=8). Data are presented as box plots displaying the median [IQR]. Statistical significance was determined using the Wilcoxon rank-sum test (ns, not significant).

Supplementary Figure S7

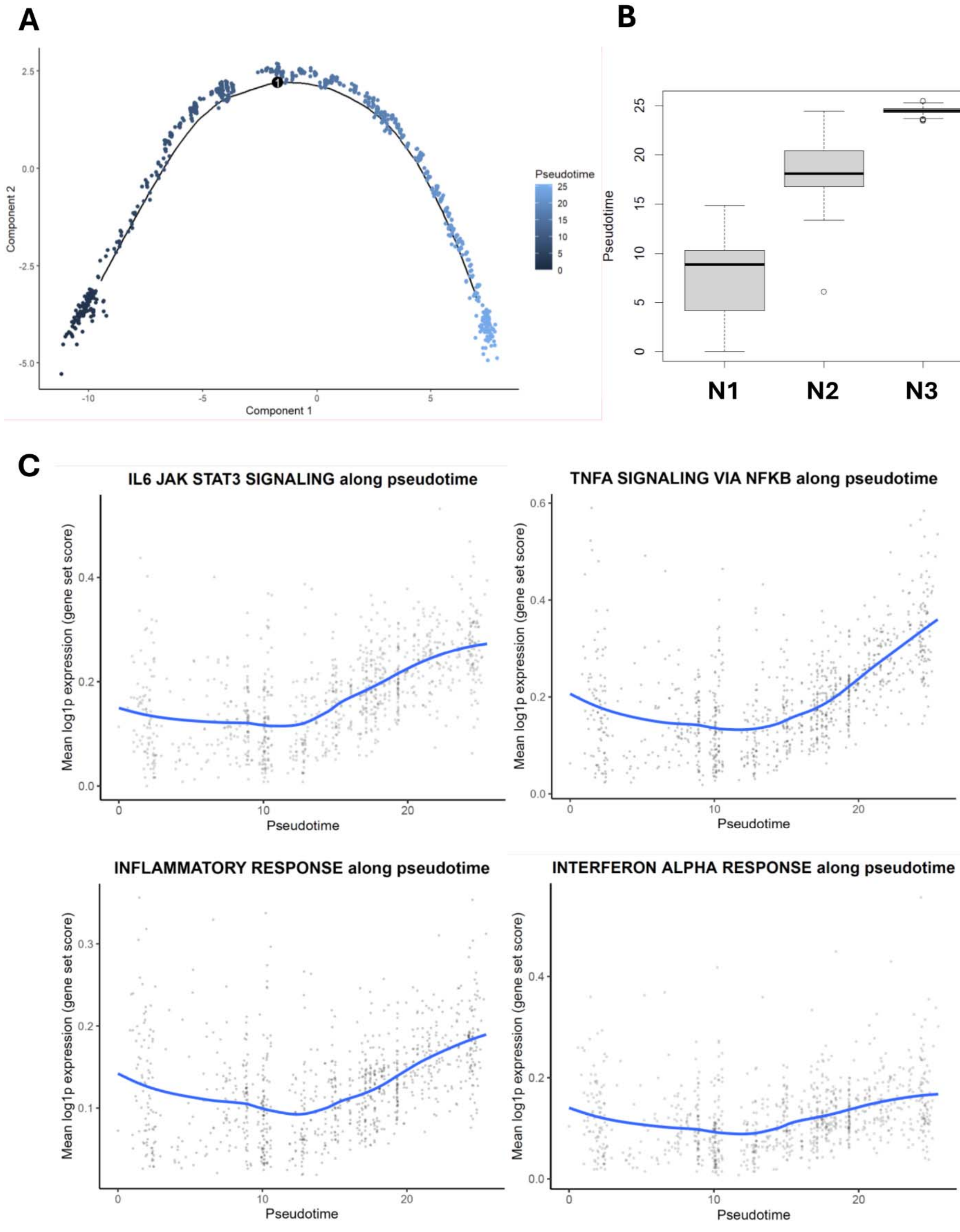

Figure S7. Pseudotime trajectory reveals the progressive inflammatory maturation of neutrophil subclusters. (A) Pseudotime trajectory plot of single-cell transcriptomes ordered along

a developmental continuum, colored by computationally inferred transcriptional states (B) Relative proportion of each defined transcriptional state within the N1, N2, and N3 subclusters. (C) Hallmark pathway enrichment scores across pseudotime.

Supplementary Figure S8

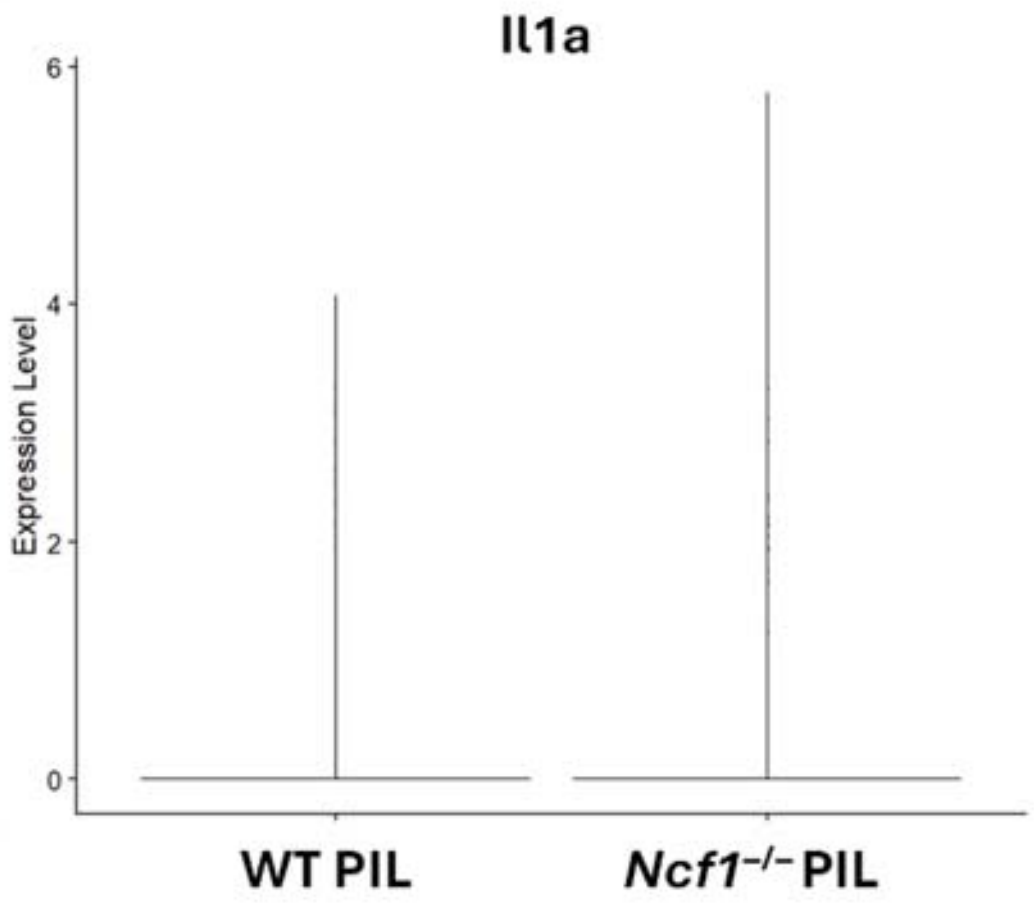

Figure S8. Transcriptional expression of *Il1a* in renal neutrophils of wild-type and NOX2-deficient PIL mice. Violin plot illustrates the distribution of *Il1a* transcript levels across the indicated groups.

Supplementary Figure S9

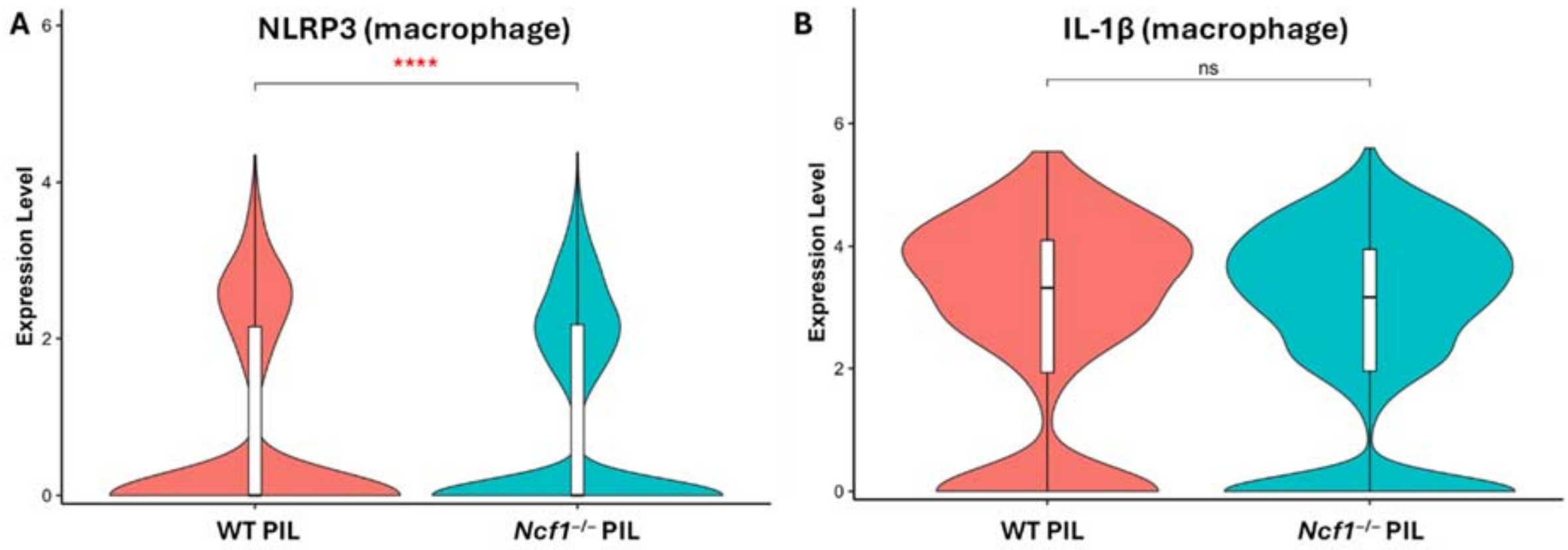

Figure S9. Transcriptional expression of *Nlrp3* and *Il1b* in renal macrophages and neutrophils of wild-type and NOX2-deficient PIL mice. Violin plots illustrate the distribution of transcript levels across the indicated groups: *Nlrp3* in macrophages (A), *Il1b* in macrophages (B). Data are presented as the median and IQR. Statistical significance was determined using the Wilcoxon rank-sum test (****p < 0.0001; ns, not significant).

Supplementary Figure S10

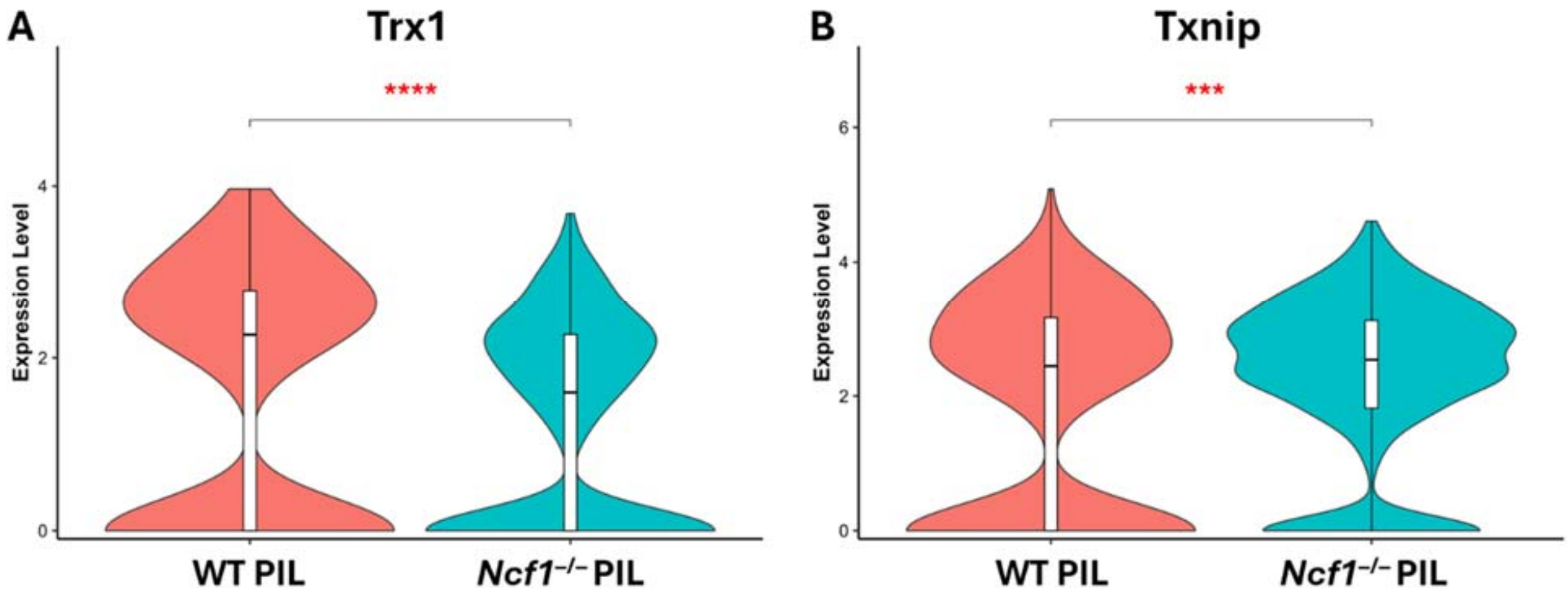

Figure S10. NOX2 deficiency drives reciprocal dysregulation of the *Txnip–Trx1* axis in renal neutrophils. Violin plots displaying the normalized expression levels of (A) *Trx1* and (B) *Txnip* in wild-type and *Ncf1*$^{-/-}$ renal neutrophils. Data are represented as median with IQR. Significance was determined using the Wilcoxon rank-sum test (**** $p < 0.0001$, *** $p < 0.001$).
