# Supplementary material for: NOX2 deficiency exacerbates lupus nephritis through activation of NLRP3 inflammasome in neutrophils
Source: J Biomed Sci. 2026 Aug 19;33:84. doi: 10.1186/s12929-026-01287-2 (PMC13491832; doi:10.1186/s12929-026-01287-2)
Supplement: Supplementary file 2 [file 12929_2026_1287_MOESM2_ESM.pdf]

Supplementary Material and Methods

1. Immunofluorescence staining

To detect endogenous IgG deposition, 5 μm formalin-fixed paraffin-embedded mouse kidney sections were deparaffinized in xylene for three times, followed by rehydration through 100% ethanol and 95% ethanol, with two times for each concentration. Antigen retrieval was achieved by heat treatment in citrate buffer at pH 6.0, followed by cooling to room temperature. Sections were then incubated with a protein blocking solution for 1 hour at room temperature to reduce non-specific binding and background and subsequently incubated overnight at 4°C with Alexa Fluor 488-conjugated goat anti-mouse IgG antibody (1:200, Cat#A-11001, ThermoFisher). On the following day, nuclei were counterstained with stained 4',6-diamidino-2-phenylindole (1:3000, DAPI; Cat#H1398, ThermoFisher) for 3min at room temperature. Slides were washed three times for 5 min each in TBS and mounted using DAKO fluorescence mounting medium (S3023, DAKO). Fluorescence images were acquired using a Zeiss microscope. For each sample, one to two glomeruli per section were evaluated at 200× or 400× magnification.

2. Bulk RNA-sequencing and data analysis

For bulk RNA-sequencing, renal neutrophils were sorted from Percoll-enriched renal leukocytes on a MoFlo XDP cell sorter (Beckman Coulter) using anti-mouse CD45 PerCP/Cy5.5 (30F11, 103130, BioLegend), CD11b PE (M1/70, 557397, BD), and Ly6G BV510 (1A8, 127633, BioLegend) antibodies. Sorted renal neutrophils were collected for RNA extraction using the RNeasy Micro Kit (QIAGEN, REF. 74004). Full-length cDNA was generated from low-input RNA using the SMART-Seq v4 Ultra Low Input RNA Kit for Sequencing (Takara, Cat. 634895). Libraries were constructed using the Nextera XT DNA Library Prep Kit (Illumina, Cat. 15032350), size-selected to 300–500 bp, and purified using AMPure XP beads (Beckman Coulter). Library concentration was quantified using Qubit fluorometer (Thermo Fisher Scientific, Cat. Q32854), and library quality was assessed

by capillary electrophoresis. Paired-end sequencing (2 × 75 bp) was performed on an Illumina NextSeq 500. Gene-level counts were generated using HTSeq, and differential expression analysis was performed using DESeq2.Genes with log2 fold change ≥ 2 and adjusted P value < 0.05 in comparisons between *Ncf1*$^{-/-}$ and WT neutrophils were considered differentially expressed for downstream visualization and pathway analyses. Rlog-transformed expression values were used for PCA and heatmap visualization, and DESeq2-normalized counts were used for volcano plots.

3. Cell-type annotation of scRNA-seq

Cell identities were annotated using a combination of canonical marker gene expression and reference-based prediction. Major immune cell populations were initially assigned at the cluster level using established lineage markers and visualized by DotPlot. Macrophages were identified by expression of Adgre1 (F4/80), *Itgam, Csf1r,* and *Mrc1*; monocytes by *Itgam* and *Ly86*; neutrophils by *S100a8, S100a9, Csf3r, Retnlg,* and *Ly6g*; dendritic cells by *Itgax, Flt3, Siglech*, and *Cd209a*; NK cells by *Ncr1, Nkg7, Gzma,* and *Ccl5*; ILC2 by *Gata3* in the absence of T-cell receptor gene expression; T cells by *Cd3d, Cd3e, Cd3g, Cd247*, and *Trbc2*; and B cells by *Cd19, Ms4a1, Cd79a,* and *Cd79b*.Reference-based annotation was additionally performed using the SingleR package with the MouseRNAseqData reference from the celldex package, using label.main as the reference label. Final cell-type labels were assigned by integrating SingleR predictions with canonical marker expression patterns, followed by manual curation in cases of ambiguity or discordance.

4. Primer sequences for quantitative Real-Time PCR

IL-1β

5’-AAGCTCTCCACCTCAATGGAC-3’ (forward)

5’-TTGGGATCCACACTCTCCAGC-3’ (reverse)

NLRP3

5’-GATCTTGGCGATCTGTGCGT-3’ (forward)

5’-CTTCCACGCCTACCAGGAAAT-3’ (reverse)

Casp-1

5’-TTTAGGCTACAGATGCCCACT-3’ (forward)

5’-GCATCATCTAAGGAAGTATTGGC-3’ (reverse)

β-actin

5’-TGGAATCCTGTGGCATCCAT-3’ (forward)

5’-AAACGCAGCTCAGTAACAGT-3’ (reverse)

5. Neutrophil subgroup analysis of single-cell RNA-seq data

The neutrophil population was extracted from the integrated dataset for subgroup analysis. Gene expression values were normalized to total UMIs per cell using the NormalizeData function. Highly variable genes were identified by the FindVariableFeatures function, followed by data scaling using the ScaleData function. Principal component analysis was performed using the RunPCA function. A shared nearest-neighbor graph was constructed using the FindNeighbors function, followed by clustering with the FindClusters function at a resolution of 0.3. Dimensionality reduction by Uniform Manifold Approximation and Projection was performed using the RunUMAP function. To remove potential contaminating populations, subclusters expressing non-neutrophil lineage markers were excluded, and the remaining cells were reprocessed using the same workflow, followed by reclustering at a resolution of 0.5. Cluster marker genes were identified using the FindAllMarkers function, with average log2 fold change ≥ 0.25 and minimum expression percentage ≥ 0.1. Differential expression analysis between KOP and WTP neutrophils was performed using FindMarkers and visualized by volcano plots. Canonical neutrophil marker genes were examined to confirm neutrophil identity, and their expression patterns were visualized using violin plots and dot plots. Hallmark gene set enrichment analysis was performed using the clusterProfiler package with

MSigDB Hallmark gene sets obtained from the msigdbr package. Pathways with a positive normalized enrichment score (NES) and adjusted $p < 0.05$ were considered enriched in KOP neutrophils.

6. Trajectory and pseudotime analysis

Trajectory analysis of WTP and KOP neutrophils was performed using Monocle2 (v2.34.0). Cells were converted from Seurat to a CellDataSet using raw UMI counts with a negative binomial model, followed by estimation of size factors and dispersions. Seurat highly variable genes (n = 2,000) were used as ordering genes. Trajectories were reconstructed using DDRTree, and cells were ordered to derive pseudotime values; the root state was determined automatically and corresponded to the N1-enriched end of the trajectory. Pseudotime was visualized on the trajectory plot and compared across N1, N2, and N3 groups. Pseudotime-associated genes were ranked across the trajectory, with top genes visualized using heatmaps and gene-wise expression trends; Hallmark pathway dynamics were assessed by gene-set scoring and ranked by correlation with pseudotime.

7. Immunoblot for protein analysis

Mouse kidney cortex tissues were lysed in RIPA buffer (Millipore, Cat. 20-188), and protein concentrations were determined using a BCA Protein Assay Kit (Thermo Fisher Scientific, Cat # 23225) according to the manufacturer's instructions. Equal amounts of protein (40 µg) were separated by 8% SDS–PAGE and transferred to PVDF membranes. Membranes were blocked with 5% BSA in TBST for 1 h at room temperature and incubated overnight at 4°C with the indicated primary antibodies. After washing, membranes were incubated for 1 h at room temperature with the appropriate HRP-conjugated secondary antibodies. Immunoreactive bands were detected using enhanced chemiluminescence (ECL) reagents (EMD Millipore, Billerica, MA) and quantified using ImageJ. Band intensities were normalized to β-actin as loading control. Primary antibodies were

rabbit anti-NLRP3 (1:500; Abcam, ab263899) and mouse anti-β-actin (1:50,000; Merck, MAB1501). Secondary antibodies were HRP-conjugated goat anti-rabbit IgG (1:10,000; Jackson ImmunoResearch, Cat. 111-035-003) and HRP-conjugated goat anti-mouse IgG (1:10,000; Jackson ImmunoResearch, Cat. 115-035-003).

8. Immunofluorescence staining for NET formation

Bone marrow neutrophils were isolated using a discontinuous Percoll gradient. Briefly, femurs and tibias were harvested, and bone marrow was flushed with serum-free RPMI, dissociated by repeated pipetting, and filtered through a 40-μm mesh. Red blood cells were lysed with ACK lysis buffer, quenched with 1× HBSS, and washed. Cells were resuspended in HBSS and layered onto a discontinuous Percoll gradient (52%, 62%, and 72%), followed by centrifugation at room temperature with low brake. Neutrophils were collected from the 62%–72% interface, washed, and seeded at 2 × 10^5 cells per well on 0.01% poly-L-lysine–coated plates, then allowed to adhere for 1 h at 37°C. Cells were stimulated with or without PMA 100 nM (Sigma, Cat. P8139) for 3 h, washed with PBS, fixed with 4% paraformaldehyde for 20 min, permeabilized with 0.2% Triton X-100 in PBS for 10 min, and blocked with 10% FBS in PBS for 1 h at room temperature. Cells were incubated overnight at 4°C with anti-MPO antibody (1:100; Abcam, ab208670) and anti–citrullinated histone H3 antibody (1:250; Abcam, ab5103), followed by incubation with an appropriate secondary antibody for 1 h at room temperature. Nuclei were counterstained with Hoechst 33258 (1:3000; Thermo Fisher Scientific, Cat. H1398) for 10 min, and cells were washed with PBS prior to imaging.

NET formation was quantified in six randomly selected, non-overlapping fields per sample. Cells exhibiting decondensed or extracellular Hoechst-positive chromatin associated with MPO and CitH3 staining were classified as NET-releasing neutrophils. The percentage of NET-releasing cells

was calculated by dividing the pooled number of NET- releasing neutrophils by the total number of neutrophils across the six fields.

Supplementary Table 1. List of antibodies used for flow cytometry analysis

**Antibodies**	**Identifier**	**Source**
CD45 (PerCP; 30-F11)	Cat. 103130	BioLegend
CD11b (PE; M1/70)	Cat. 557397	BD
Ly6G (BV510; 1A8)	Cat. 127633	BioLegend
Ly6C (FITC; HK1.4)	Cat. 128005	BioLegend
F4/80 (BV421; BM8)	Cat. 123131	BioLegend
CD64 (AF647; X54-5/7.1)	Cat. 558539	BD
pro-IL-1β (FITC; NJTEN3)	Cat. 11-7114-80	Thermo Fisher Scientific
